# Circulating and Hepatic BDCA1+, BDCA2+, and BDCA3+ Dendritic Cells Are Differentially Subverted in Patients With Chronic HBV Infection

**DOI:** 10.3389/fimmu.2019.00112

**Published:** 2019-02-04

**Authors:** Laurissa Ouaguia, Vincent Leroy, Tania Dufeu-Duchesne, David Durantel, Thomas Decaens, Margaux Hubert, Jenny Valladeau-Guilemond, Nathalie Bendriss-Vermare, Laurence Chaperot, Caroline Aspord

**Affiliations:** ^1^Institute for Advanced Biosciences, Immunobiology and Immunotherapy in Chronic Diseases, Inserm U 1209, CNRS UMR 5309, Université Grenoble Alpes, Grenoble, France; ^2^Etablissement Français du Sang Auvergne-Rhône-Alpes, R&D Laboratory, Grenoble, France; ^3^Université Grenoble Alpes, Grenoble, France; ^4^CHU Grenoble Alpes, Hepato-gastroenterology Unit, Grenoble, France; ^5^Institute for Advanced Biosciences, Research Center Inserm U1209/CNRS 5309/UGA, Analytic Immunology of Chronic Pathologies, La Tronche, France; ^6^Univ Lyon, Université Claude Bernard Lyon 1, INSERM 1052, CNRS 5286, Centre Léon Bérard, Centre de Recherche en Cancérologie de Lyon, Lyon, France

**Keywords:** hepatitis B virus, cDC1/BDCA3, cDC2/BDCA1, pDCs/BDCA2, antiviral immune responses, viral immune evasion, chronic hepatitis B patients, Type I and Type III interferons

## Abstract

**Background and aims:** Chronic hepatitis B virus (HBV) infection is a major health burden potentially evolving toward cirrhosis and hepatocellular carcinoma. HBV physiopathology is strongly related to the host immunity, yet the mechanisms of viral evasion from immune-surveillance are still misunderstood. The immune response elicited at early stages of viral infection is believed to be important for subsequent disease outcome. Dendritic cells (DCs) are crucial immune sentinels which orchestrate antiviral immunity, which offer opportunity to pathogens to subvert them to escape immunity. Despite the pivotal role of DCs in orientating antiviral responses and determining the outcome of infection, their precise involvement in HBV pathogenesis is not fully explored.

**Methods:** One hundred thirty chronically HBV infected patients and 85 healthy donors were enrolled in the study for blood collection, together with 29 chronically HBV infected patients and 33 non-viral infected patients that were included for liver biopsy collection. In a pioneer way, we investigated the phenotypic and functional features of both circulating and intrahepatic BDCA1+ cDC2, BDCA2+ pDCs, and BDCA3+ cDC1 simultaneously in patients with chronic HBV infection by designing a unique multi-parametric flow cytometry approach.

**Results:** We showed modulations of the frequencies and basal activation status of blood and liver DCs associated with impaired expressions of specific immune checkpoints and TLR molecules on circulating DC subsets. Furthermore, we highlighted an impaired maturation of circulating and hepatic pDCs and cDCs following stimulation with specific TLR agonists in chronic HBV patients, associated with drastic dysfunctions in the capacity of circulating DC subsets to produce IL-12p70, TNFα, IFNα, IFNλ1, and IFNλ2 while intrahepatic DCs remained fully functional. Most of these modulations correlated with HBsAg and HBV DNA levels.

**Conclusion:** We highlight potent alterations in the distribution, phenotype and function of all DC subsets in blood together with modulations of intrahepatic DCs, revealing that HBV may hijack the immune system by subverting DCs. Our findings provide innovative insights into the immuno-pathogenesis of HBV and the mechanisms of virus escape from immune control. Such understanding is promising for developing new therapeutic strategies restoring an efficient immune control of the virus.

## Introduction

Infection with Hepatitis B virus (HBV) is a major health problem affecting around 3.5% of the world population (1). HBV is a double stranded DNA virus which specifically infects hepatocytes and can cause chronic liver diseases (2, 3). The natural history of HBV infection results from complex interactions between the replicating virus and host's immune system (4). Whereas, patients who clear the infection elicit potent viral antigens specific T cell-mediated and humoral responses, patients who evolve toward chronicity display weak and inappropriate responses (5, 6). The physiopathology of HBV is strongly related to the host immunity, yet the mechanisms of modulation of the immune system by the virus are still misunderstood. Initiation of an effective antiviral immune response appears to be crucial for the resolution of HBV infection. However, the early steps in the recognition of the virus by immune cells and the functional consequences of this interaction remained to be studied.

A pivotal involvement of dendritic cells (DCs) is expected due to their crucial role in orchestrating antiviral immunity. Indeed, DCs are able to detect viruses and their components through multiple pattern recognition receptors (PRR), to subsequently produce large amount of antiviral cytokines especially type I and type III interferons (IFNs), and to cooperate with other immune effectors through immune checkpoints. DCs have a unique ability to uptake antigens, perform cross-presentation and prime virus-specific cytotoxic T cells (7–9). There are specialized DC subsets that differ in ontology, localization, surface marker expression, molecular phenotype, cytokine production and antigen-processing and presentation capacity (10): myeloid or conventional dendritic cells (cDCs) subdivided into two subsets based on the differential expression of CD1c/BDCA1 (cDC2) and CD141/BDCA3 (cDC1) molecules (10), and plasmacytoid dendritic cells (pDCs) expressing BDCA2 marker (9). Each DC subset displays its own repertoire of toll like receptors (TLRs), underlying their functional specialization (7, 9). TLR7 and TLR9 are mainly expressed in pDCs, while cDCs mostly express TLR3, TLR4, and TLR8 (7). TLRs stimulation triggers the expression of co-activation molecules on DCs, and the production of pro-inflammatory and anti-viral cytokines that can inhibit the viral infection and modulate innate and adaptive anti-viral immunity (8). The high plasticity of DCs allows them to orientate responses toward immunity or tolerance, depending on surrounding signals, which offer to pathogens opportunity to subvert them to escape immunity (4).

cDC2 represent the main blood DC population, whereas cDC1 are enriched within hepatic DCs (11). cDC1 are the most potent producers of IFN-λ in response to viruses or synthetic RNA polyI:C that induces TLR3 signaling (12, 13) and are specialized in antigen cross-presentation, therefore actively participating in the control of hepatotropic viruses (10, 14, 15). In the context of chronic HBV infection, functional perturbations in DCs have been described(16–18), potentially through HBsAg (19), HBcAg (20), or HBeAg (21) viral antigens. It has been shown that cDC2 can take-up HBsAg, the main envelop glycoprotein present on HBV infectious particles (22). The effect of HBsAg on cDC2 purified from healthy donors (HD) is controversial, contributing to DC dysfunction (22) or driving their strong activation in a TLR4- and CD14-dependent manner (19). Yet, few studies showed that circulating cDC2 from HBV patients displayed impairment in their maturation associated with a defective IL-12 production upon stimulation (16, 17, 22). Viral particles (23) or HBs/HBc viral antigens have also been found within pDCs from chronic HBV patients (24), suggesting direct interactions between HBV and pDCs. Decreased frequencies and functional impairment of circulating pDCs from chronic HBV patients have been reported (17, 24), as well as inhibition of pDCs from HD by HBV virus and HBsAg (21). We previously reported *ex-vivo* modulations of CD40 and CD86 expression on circulating and intrahepatic pDCs from chronic HBV patients compared to HD (25), associated with an altered OX40L expression and reduced IFNα production in response to TLR9 triggering leading to a defective triggering of NK cytotoxic effectors (25). Alterations of pDC functions in HBV patients could be linked to the binding of HBsAg to BDCA2 (21) or to the impairment of TLR9 expression (23, 24).

cDC1 are prominently present in HBV infected liver (13). Few studies showed controversial impacts of IFNλ on HBV replication in cell lines and mouse studies(14, 26), but others revealed that PEG-IFNλ induced a reduction of HBV replication in HBeAg-positive patients (27), suggesting that this cytokine may be valuable to fight chronic HBV infection. In addition, A. Woltman reported an impaired maturation together with reduced IFNλ1 production by blood cDC1 from chronic HBV patients after TLR3 triggering (13). However, the phenotype and function of both circulating and intrahepatic DCs from HBV patients has not been extensively studied, as well as the correlation of these alterations with the patient's clinical parameters. Furthermore, it is still unknown whether HBV impacts liver BDCA3+ cDC1 features.

Despite the crucial role of DCs in orientating antiviral responses and determining the outcome of infection, their precise involvement in HBV pathogenesis is not fully understood. In this study, we investigated how in humans, chronic HBV infection affects the functions of both blood and liver cDC2, pDCs, and cDC1, by analyzing their frequency, basal activation status, expression of specific immune checkpoints and TLR molecules, and their ability to secrete a large panel of cytokines including IFNs and IFNλs in response to specific TLR stimulations. We also assessed the clinical relevance of these modulations. Our findings highlighted major alterations of DC's phenotype and function in chronic HBV patients, suggesting deep impairments of the innate immune response. This study demonstrates that HBV may subvert DCs to escape immunity and bring insights into the mechanisms of virus escape from immune control. Such understanding may be promising for developing new therapeutic strategies restoring an efficient immune control of the virus.

## Materials and Methods

### Patient and Control Samples

This protocol conformed to the ethics committee of Grenoble University Hospital (CHU-Grenoble) and the French Blood Service's (EFS-AuRA) Institutional Review Board and was declared under the number DC-2008-787 and DC-2011-1487. Written informed consent was obtained from all participants prior to their enrolment in this study. Blood samples were obtained from chronically HBV infected patients (HBV, *n* = 130) and healthy donors (HD, *n* = 85). Exclusion criteria included: infection with human immunodeficiency virus, co-infection with hepatitis C or D virus, other liver diseases, and current treatment with IFNα or immunosuppressive agents. Peripheral blood mononuclear cells (PBMCs) were isolated from heparinized blood samples using Ficoll-paque density gradient centrifugation according to the manufacturer's instructions (Eurobio). Plasma samples were collected and stored frozen. Serum HBsAg and viral load (HBV DNA) levels were quantified using the Abbott Architect i2000sr-QT assay (Abbott) and COBAS Ampliprep/Taqman (Roche), respectively. Liver biopsy samples were obtained from 29 HBV-infected patients and 33 non-viral infected patients. Liver tissue was reduced to cell suspensions by mechanical disruption. The clinical characteristics of the patients are summarized in Supplementary Table 1.

### Flow Cytometry Phenotypic Analysis

Fresh PBMCs were stained with fluorochrome-labeled anti-human CD11c, CD86, HLA-DR, OX40L, 4-1BBL (BD), Lin (Biolegend), CD40, CD45, CD80, CD1c/BDCA1, TLR4 (Beckman), 4-1BBL (Clinisciences), ICOS-L/CD275, TLR9 (eBiosciences), BDCA2, BDCA3 (Miltenyi), and GITRL (R&D systems) antibodies. TLR3 (Abcam), TLR8 (Novus) and TLR9 (eBiosciences) staining were performed using anti-human TLR after surface molecules staining and cell permeabilization. Fresh liver cell suspension (LMNCs) were stained with anti-human CD11c, CD86, HLA-DR (BD), Lin (Biolegend); CD40, CD45, CD80, CD1c/BDCA1 (Beckman), BDCA2 and BDCA3 (Miltenyi) antibodies. Stained cells were then analyzed using LSRII Flow Cytometer and FACSDiva software (BD). Isotype controls were used to discriminate positive cells from nonspecific background staining and dead cells were excluded with Live and Dead cell stain (ThermoFisher). Mean fluorescence intensity (MFI) was analyzed and shown only when the mean percentage of total positive cells was ≥30%. To ensure quality control during the study, we performed a systematic standardization of the fluorescence intensities using cytometer setup and tracking beads (BD).

### Functional Analysis of Circulating and Intrahepatic DCs in Response to TLRs Triggering

#### Intracellular Cytokine Staining

For intracellular cytokine characterization, 500 μl of fresh whole blood of HD or chronic HBV patients were cultured for 5 h with or without TLRs ligands alone or mixed together, comprising polyinosinic-polycytidylic acid (polyI:C, 100 μg/mL), Imiquimod (R848, 1 μg/mL) and Class-A CpG oligonucleotide ODN-2336 (CpG_A_, 50 μg/mL) (Invivogen). 1 μg/mL of Brefeldin A (BD) was added for the last 4 h. Subsequently, cells were stained for surface molecules with fluorochrome-labeled anti-human CD11c, HLA-DR (BD), Lin (Biolegend), CD45, CD1c/BDCA1(Beckman), Live and Dead (ThermoFisher), BDCA2 and BDCA3 (Miltenyi) antibodies. Cells were then fixed and permeabilized for intracellular cytokine staining using the fluorochrome-labeled anti-human TNFα, IL-12p40/70 (BD), IFNα (Miltenyi) antibodies and anti-human IFNλ1 (Novus) stained with mix-n-stain CF488 (Biotum). Cytokine-producing cell frequencies were analyzed by flow cytometry using LSRII Flow Cytometer instrument and FACSDiva software (BD).

#### Maturation and Cytokine Secretion

To analyze the maturation of DCs upon TLR triggering, freshly isolated PBMCs and LMNCs from chronic HBV patients or HD/non-viral infected controls were cultured at 1 × 10^6^cells/mL for 22 h with or without a single or a mixture (MIX) of TLRLs comprising polyI:C (30 μg/mL), R848 (1 μg/mL) and CpG_A_ ODN-2336 (1 μmole/L) (Invivogen). All cultures were performed in RPMI-1640/GlutaMAX (Invitrogen) supplemented with 1% non-essential amino acids, 100 μg/mL gentamycin, 10% fetal calf serum (Invitrogen), and 1 mmol/L sodium pyruvate (Sigma). Peripheral and hepatic DC's activation status was measured by flow cytometry using fluorochrome-labeled anti-human CD11c, CD86, HLA-DR (BD), Lin (Biolegend), CD40, CD45, CD1c/BDCA1, CD80 (Beckman), Live and Dead (ThermoFisher), BDCA2, and BDCA3 (Miltenyi) antibodies. Analysis was performed using LSRII Flow Cytometer and FACSDiva software (BD). PBMCs and LMNC supernatants were harvested after 22 h of culture and IL-12p70, IFNα2, IFNβ, IFNλ1, IFNλ2, TNFα, TGFβ1, IP10, and MCP-1 cytokine secretions were measured by Luminex Technology according to manufacturer protocol using MAGPIX®200 Instrument with xPONENT® software (Bio-Rad). Inter-assay variability was determined by quantifying the same control sample each time, which was frozen in multiple aliquots thawed once the day of the assay. Cytokine secretion was reported to corresponding major DC subsets by calculating the amount/10^5^DC number.

## Statistical Analysis

Statistical analyses were performed using Mann–Whitney non-parametric *U-*test, 2-way row matching analysis of variance (2-way-RM-ANOVA) and Spearman correlation using Graph Pad Prism software version 5.01 (Graph Pad).

## Results

### Circulating and Intrahepatic BDCA1+ cDC2, BDCA2+ pDCs, and BDCA3+ cDC1 From Chronic HBV-Infected Patients Display an Altered Frequency

**Figure 1:**
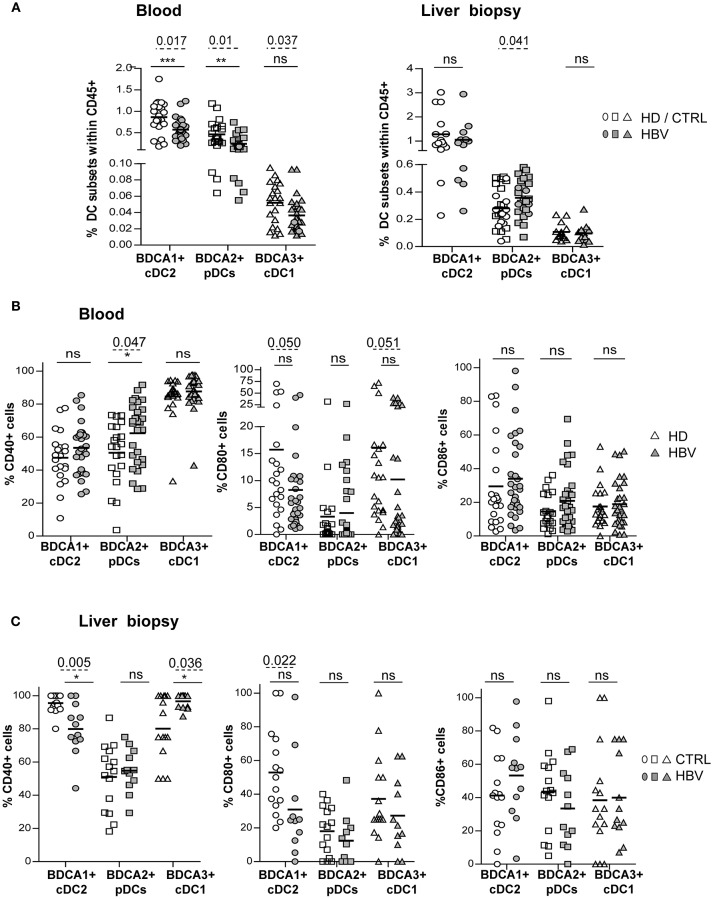
Peripheral and intrahepatic DC subsets from chronic HBV patients displayed modulation of their basal activation status. DC subset's frequencies and expression of co-activation molecules CD40, CD80, and CD86 were analyzed by flow cytometry on blood and liver DCs of chronic HBV patients and HD or non-viral infected controls. Frequency analyses of BDCA1+ cDC2, BDCA2+ pDCs, and BDCA3+ cDC1 within **(A)** PBMCs and LMNCs among living CD45+ cells. Open symbols, HD or non-viral infected controls (CTRL) (blood, *n* = 19–21; liver, *n* = 27 for pDCs, and *n* = 15 for cDCs); filled symbols, chronically HBV-infected patients (HBV) (blood, *n* = 28–30; liver, *n* = 25–27 for pDCs and *n* = 11 for cDCs). **(B,C)** Expression levels of the co-activation molecules CD40, CD80, and CD86 on **(B)** circulating and intrahepatic **(C)** cDC2, pDCs and cDC1 [**(B)**, open symbols, HD (*n* = 21); filled symbols, HBV (*n* = 29–31) or **(C)** open symbols, controls (*n* = 15); filled symbols, HBV (*n* = 11–12)]. Results are expressed as percentages of positive cells. Bars indicate mean. *P*-values were calculated using the 2-way-RM ANOVA test (straight line, *P ≤ 0.05, **P < 0.01, ***P < 0.001) and Mann–Whitney test (dashed lines).

We designed a multiparametric flow cytometry strategy allowing the simultaneous and extensive analysis of the phenotype of the three major DC subsets, from blood and liver samples (Supplementary Figure S1A). Among CD45+ cells within fresh PBMCs or LMNCs, BDCA1+ cDC2 were defined as Lin-HLADR+CD11c+CD1c/BDCA1+ cells, pDCs identified as Lin-HLADR+CD11c-BDCA1-BDCA2+ cells, and BDCA3+ cDC1 picked out as Lin-HLADR+CD11c+BDCA3^high^ cells (Supplementary Figure S1A). Evaluation of the proportion of cDC2, pDCs and cDC1 highlighted significant reduced frequencies and absolute number for circulating cDC2 and pDCs in addition to a reduced frequency of cDC1. Moreover, results showed a higher frequency of pDCs in the liver from chronic HBV patients compared to HD (Figure 1A; Supplementary Figure S1B) while similar percentages of liver cDCs were observed, suggesting a specific active recruitment of blood pDCs to HBV-infected livers. Interestingly, the frequency of circulating cDC2 and intrahepatic pDCs correlated with HBsAg levels, negatively and positively, respectively (Supplementary Figure S1C), suggesting that HBV may modulate DC's relative repartition between blood and liver. In addition, frequencies of circulating and intrahepatic DCs were also correlated between them in chronic HBV patients (Supplementary Figures S1D, S1E), suggesting that all DCs were modulated simultaneously.

### The Modulated Expression of Immune Checkpoints and TLRs by Circulating DCs From Chronic HBV Patients Correlates With HBV DNA and HBsAg Levels

We next investigated the basal activation status of peripheral and intrahepatic DCs (Figures 1B,C; Supplementary Figures S2A,B,C). Analyses revealed that blood and liver cDC2 from HBV patients displayed reduced expression of CD40 and/or CD80 (Figures 1B,C) indicating that cDC2 in HBV patients are less activated compared to HD. In contrast, circulating and liver pDCs (Figure 1B; Supplementary Figure S2C) displayed an upregulation of CD40 suggesting that HBV infection could also modulate pDC activation status. Furthermore, we observed a lower expression of CD80 on circulating cDC1 in HBV patients while intrahepatic cDC1 showed higher CD40 expression (Figures 1B,C), which correlated positively with HBsAg levels (Supplementary Figure S2D). This suggests that circulating cDC1 with an immature status become activated when localizing within HBV-infected livers. Interestingly, CD80 expression correlated between peripheral and intrahepatic cDC2 and cDC1 (Supplementary Figures S2E,F) in chronic HBV patients, suggesting that HBV modulates simultaneously CD80 expression on DCs. Besides, CD40 expression on intrahepatic pDCs was negatively correlated with HBV DNA and HBsAg levels (Supplementary Table 6), pointing out the clinical relevance of the modulation of basal DCs's status.

**Figure 2:**
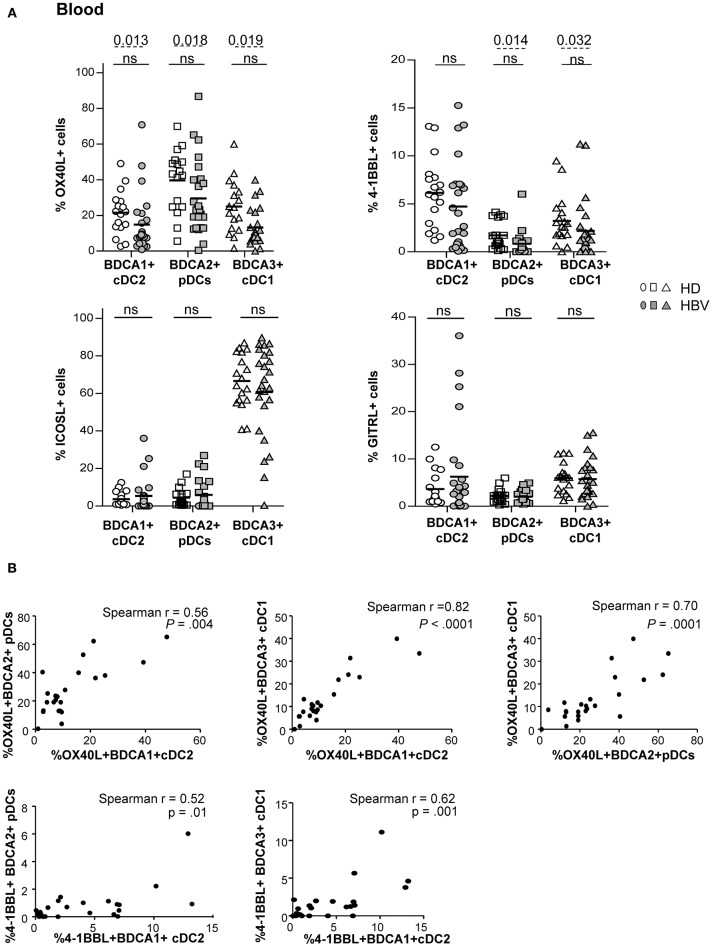
Impaired OX40L and 4-1BBL co-stimulatory molecules expression on peripheral DC subsets from chronic HBV patients. Co-stimulatory molecules expression was determined by flow cytometry on peripheral DC subsets among fresh PBMCs isolated from HD and HBV patients. **(A)** Percentages of cDC2, pDCs, and cDC1 expressing OX40L, 4-1BBL, ICOSL, and GITRL within PBMCs. Open symbols, HD (*n* = 17–18); filled symbols, HBV (*n* = 23–25). Bars indicate mean. *P*-values were calculated using the 2-way-RM ANOVA test (straight line) and the Mann-Whitney test (dashed lines). **(B)** Spearman's correlations of OX40L and 4-1BBL expression between peripheral cDC2, pDC, and cDC1 subsets in chronic HBV patients (*n* = 29–31).

As DCs are crucial in immunity induction, we further assessed the expression of co-stimulatory/co-inhibitory molecules involved in DC's cross-talks with immune effectors, including OX40L, 4-1BBL, GITRL, ICOSL, and PDL1 (Supplementary Figure S3A). The percentages of expression of OX40L and 4-1BBL co-stimulatory molecules were significantly down-regulated on peripheral pDCs and cDC1 while only OX40L was reduced on cDC2 (Figure 2A and Supplementary Figure S3B) from chronic HBV patients compared to HD. The proportion of blood (Supplementary Figure S3C) and liver DCs expressing the co-inhibitory molecule PDL1 was similar between HBV patients and controls but negatively correlated with HBV DNA in viremic patients for cDC2 and pDCs (Supplementary Tables 2, 3). Importantly, the proportion of cells expressing these immune checkpoints negatively correlated with HBsAg and/or HBV DNA in HBV patients (Supplementary Figure S3D; Supplementary Tables 2–4). Furthermore, analyses highlighted tight correlations of the expression of OX40L and 4-1BBL between peripheral cDC2, pDCs, and cDC1 in HBV patients (Figure 2B), indicating that immune checkpoint molecules on blood DCs were modulated alike. Hence, by altering the immune checkpoints expression on DCs, HBV may subvert DC cross-talks with immune cells and impair the subsequent activation of anti-viral immune effectors.

**Figure 3:**
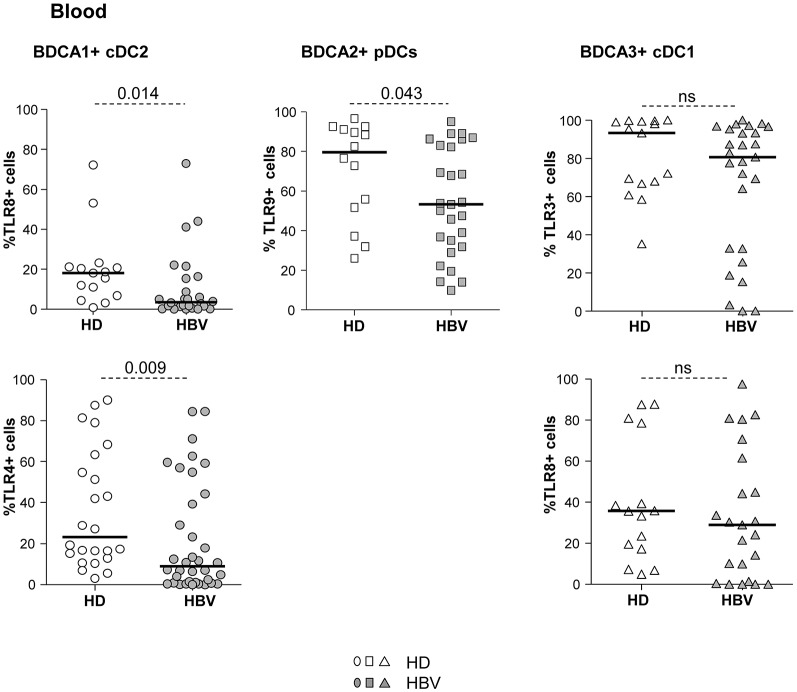
Impaired expression of TLR4, TLR8, and TLR9 in peripheral DC subsets from chronic HBV patients. Surface (TLR4) or intracellular (TLR3, TLR8, and TLR9) expression of TLRs was evaluated by flow cytometry on peripheral DC subsets from fresh PBMCs isolated from HD and HBV patients. Percentages of cDC2, pDCs, and cDC1 expressing the TLR molecules within the corresponding DC subset. Open symbols, HD (*n* = 14–15 for TLR3,8,9, and *n* = 27 for TLR4); filled symbols, patients with chronic HBV (*n* = 24 for TLR3,7,9, and *n* = 35 for TLR4). Bars indicate median. *P*-values were calculated using the Mann-Whitney test. *P*-values were calculated using Man-Whitney test (dashed lines).

To assess the influence of chronic HBV infection on DC's ability to sense pathogens through TLRs, we investigated the basal expression levels of specific TLRs on each peripheral DC subset (Supplementary Figure S4A). We observed a significant alteration in the percentages of TLR4 and TLR8 expression on cDC2, a reduced expression of TLR9 on pDCs and a tendency to a down-regulation of TLR3 expression on cDC1 expression (Figure 3; Supplementary Figure S4B) from chronic HBV patients compared to HD. These data strongly suggest that HBV impairs TLRs expression on circulating DCs which may in turn reduce their innate immune functions.

### The Maturation of Circulating and Intrahepatic cDCs and pDCs Upon TLR Triggering Is Impaired in Chronic HBV Patients According to HBsAg Levels

**Figure 4:**
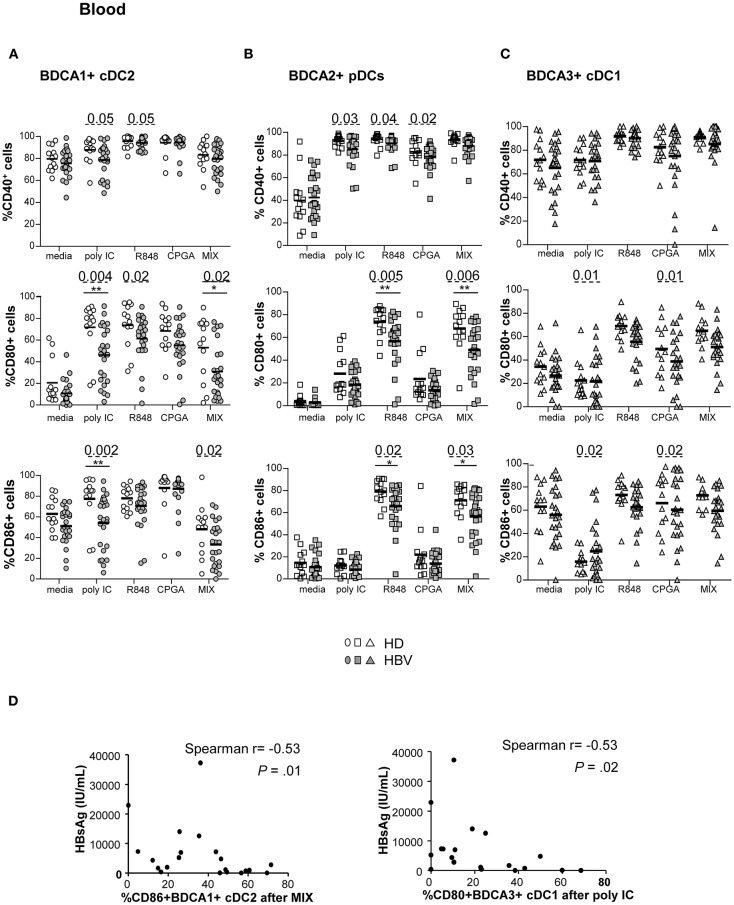
Defective maturation of circulating BDCA1+ cDC2, BDCA2+ pDCs, and BDCA3+ cDC1 from chronic HBV patients upon TLR triggering. PBMCs from HD or HBV patients were stimulated for 22 h with or without TLRLs (polyI:C, R848 or CPG_A_ (ODN2336)) alone or mixed together (MIX:polyI:C+R848+CPG_A_) and the expression of the maturation markers was measured by flow cytometry. Percentages of CD40, CD80, and CD86 molecules on **(A)** cDC2, **(B)** pDCs, and **(C)** cDC1. Open symbols, HD (*n* = 13–18); filled symbols, HBV (*n* = 22–26). *P*-values were calculated using the 2-way-RM ANOVA test (straight line) *P ≤ 0.05, **P < 0.01 and Mann–Whitney test (dashed lines). Bar indicates mean. **(D)** Spearman correlations between the expression of maturation molecule on cDC2 and cDC1 from HBV patients and plasmatic HBsAg levels (*n* = 19–21).

To understand whether the alterations of TLR expression on circulating DCs resulted in an impairment of their ability to respond to TLRL stimulation, we monitored the expression of activation molecules by cDC2, pDCs, cDC1 in response to specific single or combined TLR ligands (Figure 4; Supplementary Figure S5). The upregulation of CD40, CD80, and CD86 (% and/or MFI) was significantly hampered on circulating DCs (Figure 4; Supplementary Figure S5) from HBV patients compared to HD upon TLR3, TLR7/8, and/or TLR9 triggering. Extensive analyses showed that these modulations were positively correlated between peripheral cDC2 and pDCs together with strong cross-correlations between the activation markers in HBV patients (Supplementary Figure S6). These data indicate that the *ex-vivo* maturation of peripheral DCs after TLR triggering is altered likewise on all blood DCs in chronic HBV patients. Interestingly, further analyses indicated significant negative correlations between the impaired maturation status of cDCs from chronic HBV patients with HBsAg and HBV DNA (Figure 4D; Supplementary Tables 2,4), unlike pDCs. Hence, these data suggested that circulating cDCs from chronic HBV patients exhibit dysfunctional maturation responses to TLR stimuli that are closely linked to viral parameters.

**Figure 5:**
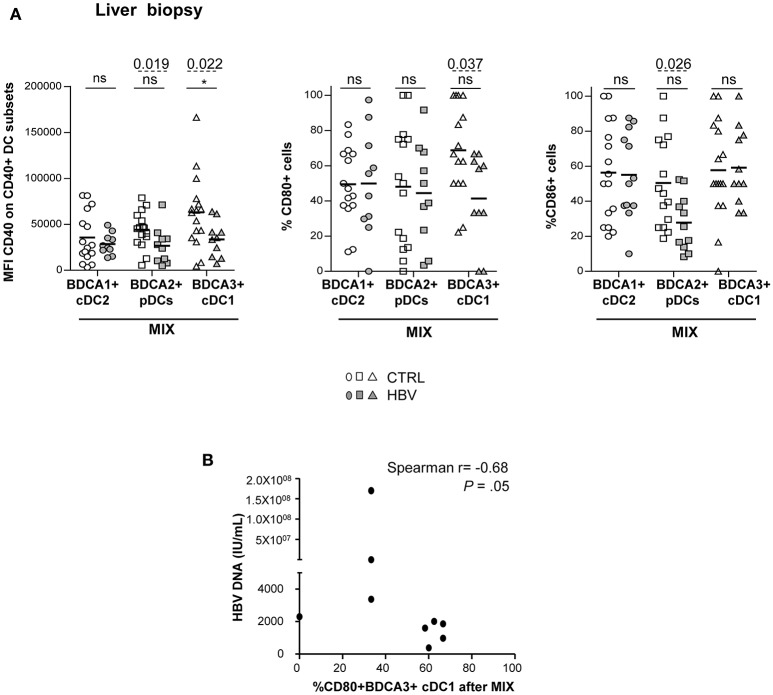
Altered maturation of intrahepatic BDCA2+ pDCs and BDCA3+ cDC1 in chronic HBV patients after TLRs stimulation. LMNC suspensions from non-viral infected controls or HBV patients were stimulated for 22 h with a mixture of TLRLs (MIX:polyI:C+R848+CPG_A_) and the expression of the maturation markers CD40, CD80, and CD86 was measured by flow cytometry. **(A)** Percentages or MFI of CD40, CD80, and CD86 molecules on cDC2, pDCs, and cDC1. Open symbols, CTRL (*n* = 15–16); filled symbols, HBV (*n* = 10–12). *P*-values were calculated using the 2-way-RM ANOVA test (straight line, *P ≤ 0.05) and Mann–Whitney test (dashed lines). Bar indicated mean. **(B)** Spearman correlations between CD80 on intrahepatic cDC1 from HBV patients after MIX stimulation with HBV DNA (*n* = 9).

Due to the limited amount of biopsy material, we assessed the ability of intrahepatic DCs to respond to TLR triggering by stimulation of LMNCs with only the mixture of TLR ligands. Noteworthy, we revealed an impairment of the upregulation of CD40 and CD86 molecules on intrahepatic pDCs, and of CD40 and CD80 markers on intrahepatic cDC1 Figure 5A from chronic HBV patients compared to controls. In addition, we showed that the impaired upregulation of CD40 and CD80 on cDC2 and cDC1 upon TLR triggering correlated with HBsAg or HBV DNA (Figure 5B; Supplementary Table 6), and the expression of CD86 after TLRs stimulation correlated between intrahepatic DCs (Supplementary Figure S7) in HBV patients. cDC2 maturation after TLR triggering was similar between chronic HBV patients and controls. Taken together, these results suggest that HBV modulates intrahepatic pDCs and cDC1's maturation in a comparative way. Hence, these results strongly suggest that HBV infection can impair DC's maturation at both peripheral and hepatic levels and that could be linked to viral parameters.

### Peripheral cDCs and pDCs From Chronic HBV Patients Display an Impaired Antiviral Cytokine Production Upon TLR Triggering That Correlates With Viral Parameters

**Figure 6:**
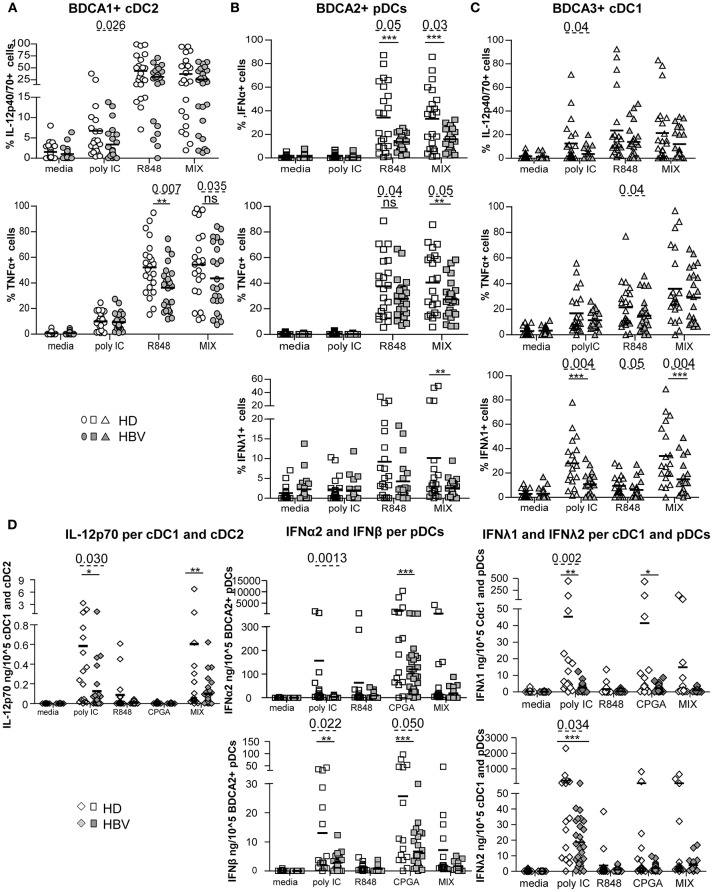
Impairment of intracellular IL-12p70, TNFα, IFNα, and IFNλ1 (IL-29) production by blood DC subsets from chronic HBV patients upon TLR triggering. Whole blood samples were stimulated for 5-h with or without polyI:C, R848, CPG_A_ (ODN2336) alone or mixed together (MIX:polyI:C+R848+CPG_A_) and the production of cytokines by each DC subset was measured by intracellular staining by flow cytometry. Percentages of cytokine-expressing cells within **(A)** cDC2, **(B)** pDCs, and **(C)** cDC1. Open symbols, HD (*n* = 20–24); filled symbols, HBV (*n* = 20–26). **(D)** Cytokine's secretion by PBMCs were reported to the absolute number of DCs present in sample before stimulation with TLRLs by calculating the cytokine production per DC subsets in each sample (amount of cytokine/10^5^DCs) for cDC2+cDC1 (IL-12p70), pDCs (IFNα2, IFNβ) and cDC1+pDCs (IFNλ1, IFNλ2). Results are expressed in ng or pg/10^5^DC subsets. Open symbols, HD (*n* = 20–24); filled symbols, HBV patients (*n* = 20–26). *P*-values were calculated using the 2-way-RM ANOVA test (straight line) *P ≤ 0.05, **P < 0.01, ***P ≤ 0.001; and the Mann–Whitney test (dashed lines).

The functional capacity of circulating DCs to produce cytokines was subsequently investigated by performing intracellular labeling of IL-12p40/70, TNFα, IFNα, and IFNλ1 stimulated or not, with single or combined TLRLs (Figure 6; Supplementary Figure S8A). We observed significant impairments of IL-12p40/70 and TNFα production by cDC2, IFNα, TNFα, and IFNλ1 production by pDCs and IFNλ1, TNFα, and IL-12p40/70 production by cDC1 from chronic HBV-infected patients compared to HD under stimulation with polyI:C and/or R848, and/or MIX agonists (Figures 6A–C). Interestingly, the proportions of cytokine-producing cells within each DC subset after TLRs stimulation were highly correlated in chronic HBV patients, as observed for IL-12p40/70- and TNFα-producing cDC2; IFNα- and TNFα-producing pDCs; and IFNλ1-, TNFα-, and IL-12p40/70-producing cDC1 (Supplementary Figure S8). Moreover, the impaired productions of anti-viral cytokines within specific peripheral DCs were closely related, as we observed positive cross-correlations between IFNα/TNFα-producing-pDCs, IL-12p40/70/TNFα-producing-cDC2, and IFNλ1-producing-cDC1 (Supplementary Figure S9). Remarkably, we noticed that impairments of peripheral DCs to produce IFNλ1, TNFα, and IL-12p40/70 were negatively correlated with HBsAg or HBV DNA from chronic HBV patients (Supplementary Figure S9 D; Supplementary Tables 2–4). All together, these data indicate that the capacity of circulating DCs to produce anti-viral cytokines upon TLR activation is highly impaired in chronic HBV-infected patients. Furthermore, we explored the secretion of a larger panel of cytokines/chemokines by Luminex in the supernatants of PBMCs stimulated or not with a single or a mixture of TLR ligands. We observed a lower production of IFNα2, IFNβ, IFNλ1, and IFNλ2 anti-viral cytokines upon stimulation with polyI:C and/or CpG_A_ TLR ligands (Supplementary Figure S10A). As we know which DC subsets are the major producers of specific cytokines, we assessed the impact of HBV on DC subsets for specific cytokines by expressing the concentration of the secreted cytokines IL-12p70 per cDC1+cDC2; IFNα2 and IFNβ per pDCs; IFNλ1 and IFNλ2 per cDC1+pDCs in the corresponding samples. Analyses confirmed an impaired secretion of those cytokines in the corresponding DC-producing them upon polyI:C, CpG_A_ and/or MIX stimulation (Figure 6D), that negatively correlated with HBV DNA in HBV patients (Supplementary Figure S10 B; Supplementary Table 5). Among the other cytokines or chemokines commonly produced by DCs, we found an overexpression of the chemokine MCP-1 upon polyI:C and CpG_A_ stimulation that positively correlated with HBsAg levels (Supplementary Figure S11). This data suggests that HBV may favor the trafficking of immune cells to infection sites. We also highlighted a significant overproduction of the immunosuppressive cytokine TGF-β1 from chronic HBV patients compared to HD under steady state that was conserved upon stimulation with TLRL (Supplementary Figure S11A). Taken together, these data indicated that HBV impairs antiviral cytokine production and increase immuno-regulatory cytokine secretion by blood DCs.

### Intrahepatic DCs From Chronic HBV Patients Are Fully Functional Upon TLR Triggering

**Figure 7:**
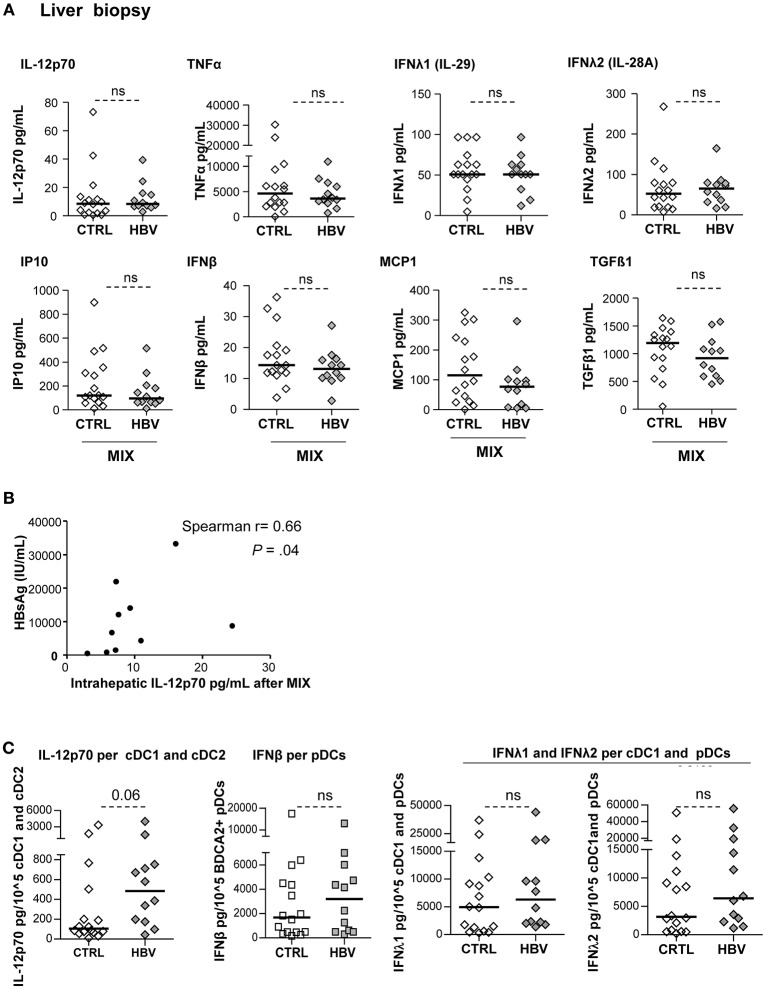
Improvement of cytokine secretion by LMNCs in chronic HBV patients upon TLR triggering. LMNCs (1 × 10^6^cells/ml) from non-viral infected controls or HBV patients were stimulated for 22 h with a mixture of TLRL (MIX:polyI:C+R848+CPG_A_) and the culture supernatants were examined for the presence of IL-12p70, IFNβ, IFNλ1, IFNλ2, TNFα, IP10, MCP1, IL-10, and TGF-ß1 by Luminex technology. **(A)** Quantification of cytokine secretion in the supernatants. Open symbols, HD (*n* = 16); filled symbols, HBV (*n* = 11–12). *P*-values were calculated using Mann–Whitney test. **(B)** Spearman correlation between the IL-12p70 secretion by intrahepatic LMNCs from HBV patients with plasmatic HBsAg levels (*n* = 10). **(C)** Cytokine's secretion by liver cell suspensions were reported to the absolute number of DCs present in samples before stimulation with the mix of TLRLs (amount of cytokine/10^5^DC) as shown for cDC2+cDC1 (IL-12p70), pDCs (IFNβ) and cDC1+pDCs (IFNλ1, IFNλ2). Open symbols, HD (*n* = 16–17); filled symbols, HBV (*n* = 28–30). *P*-values were calculated using Mann–Whitney test. *P*-values were calculated using Man-Whitney test (dashed lines).

We investigated the impact of HBV on cytokine production by LMNCs upon stimulation with a mixture of TLRL (Supplementary Figure 12; Figure 7). Results obtained on HBV samples showed that IL-12p70, TNFα, IFNβ, and IFNλ2 are not secreted in unstimulated condition but secreted upon TLRL stimulation of biopsy samples, while MCP1, TGFβ1, and IP10, that are not TLRL dependent, were present under steady state (Supplementary Figure 12A). Most of the cytokines/chemokines analyzed were similarly produced by LMNCs from chronic HBV patients and controls (Figure 7A) but some positively correlated with HBsAg levels (Figure 7B; Supplementary Table 6). IFNα2 secretion by hepatic cells was not shown because samples were below the detection limit of the assay. As previously described for PBMCs, we calculated the concentration of IL-12p70 per cDC1+cDC2; IFNβ per pDCs; IFNλ1 and IFNλ2 per cDC1+pDCs in the corresponding samples. Very interestingly, analyses highlighted a similar ability of intrahepatic DCs to secrete IL-12p70, IFNβ, IFNλ1, and IFNλ2 in patients compared to controls (Figure 7C). These results suggest that intrahepatic DCs are still functional with a higher tendency to respond to TLR triggering by secreting the critical anti-viral cytokines. Notably, the production of those cytokines upon TLR triggering within specific hepatic DCs were closely related, as we observed strong positive cross-correlations between IL-12p70-producing-cDC1+cDC2 and IFNλ2-producing cDC1+pDCs, and IFNβ-producing-pDCs and IFNλ1/IFNλ2-producing cDC1+pDCs (Supplementary Figure S12B). Hence, higher levels of pro-inflammatory cytokines in the liver following TLR activation indicate that HBV might favor liver inflammation that could lead to a worsening of the liver disease, while dampening DCs' fitness driving an impaired anti-viral immunity.

## Discussion

DCs play a central role in viral infections through their unique ability to trigger antiviral effectors and produce cytokines upon pathogens or virus-infected cell recognition. HBV develops multifaceted strategies to evade this immuno-surveillance, yet the mechanisms of this escape remains poorly investigated. We highlight here alterations in the phenotype and function of circulating BDCA1+ cDC2, BDCA2+ pDCs, and BDCA3+ cDC1 associated with modulations of intrahepatic DC subsets, revealing that HBV may subvert DCs to escape immune control and favor disease worsening. So far, most analyses have been limited to the *in vitro* study of HBV or HBV antigen impacts on peripheral DCs purified from HD, and/or in patients but shrunken to the assessment of activation markers and few cytokines. Very few studies investigated the impact of HBV on blood cDC1 and even less studies assessed the features of intrahepatic DC subsets nor even their functionality directly in patients. In the present work, we performed extensive analyses of the three major DC subsets simultaneously, directly *ex-vivo* in HBV patients, in both blood and liver. This study provides novel insights into the immuno-pathogenesis of HBV infection and help explaining the failure of the triggering of an effective anti-viral immune response.

We first showed reduced proportions of the 3 circulating DC subsets together with an increased frequency of intrahepatic pDCs in chronic HBV patients compared to controls. Perturbations of peripheral DCs have been already reported in HBV patients, especially lower frequencies of cDC2 and pDCs (24, 25). Accumulation of CD11c+ cells (28, 29), pDCs (29), and cDC1 (13) into portal areas of the liver of HBV patients were also shown by assessing the density of these cells upon immuno-histochemical staining of liver sections. We observed no difference between intrahepatic cDCs from chronic HBV patients compared to controls. Such discrepancies can be explained by analysis of large tissue section via performing multiparametric flow cytometry on LMNCs leading to a more accurate definition of DC subsets. Interestingly, our study reveals tight correlations between peripheral or intrahepatic DCs's frequencies, suggesting that HBV simultaneously affects DCs' prevalence. In addition, we observed strong negative and positive correlations between circulating cDC2's and intrahepatic pDC's frequency with plasmatic HBsAg levels, respectively. These data, together with other studies, suggest an active recruitment of pDCs from blood to the infection sites. HBV may therefore directly impact DC trafficking, although we cannot exclude other factors which may affect the circulating DC pools such as the potential direct impact of HBV/HBV-derived factors on DCs enhancing their susceptibility to apoptosis (16), or the potential reduction of DC progenitors within the bone marrow (28).

We show that circulating and/or intrahepatic cDC2 display an impaired basal expression of co-activation (CD40, CD80) and co-stimulatory (OX40L) markers associated with a reduced expression of TLR8 and TLR4 sensors. Interestingly, we also highlight impairments in their ability to mature *ex-vivo* as well as to secrete IL-12p70 and TNFα upon TLR triggering for blood cDC2 from chronic HBV patients compared to controls whereas liver cDC2 display no impairment in these capacities. These results bring additional evidences to studies describing decreased expression of co-activation molecules and defective IL-12 production by circulating BDCA1+ cells of chronic HBV patients (16, 17, 22). The observed modulations of maturation and cytokine secretion upon TLR triggering could result either from a direct effect of specific TLR on corresponding DCs or can be linked to the indirect impact of other DCs. Therewith, we described for the first time correlations between cDC2 impairments and plasmatic HBsAg and HBV DNA, indicating a direct impact of HBV and/or HBV proteins on cDC2 phenotype, maturation, and function. Indeed, it is known that liver and blood of HBV-infected individuals can reach levels of 10^9^–10^10^/mL of infectious particles and 10,000-fold higher concentrations of HBsAg (30), favoring multiple interactions with DCs. Previous data reported that HBsAg can interact *in vitro* with cDC2 from HD in a TLR4- and CD14-dependent manner (19), potentially resulting in HBsAg picking up by cDCs (22). Hence, these results suggest that HBV may hijack the anti-viral immune responses through hampering cDC2.

We previously demonstrated that pDCs from chronic HBV patients display modulations of immune checkpoint molecules in blood and liver, together with a reduction of IFNα-secretion following TLR9 triggering for blood pDCs (25). We confirmed here on a new cohort that circulating and intrahepatic pDCs from chronic HBV patients display a more activated status, a down-regulation of OX40L and 4-1BBL expression along with an alteration of TLR9 expression on circulating pDCs, and further showed a defective maturation and a reduced IFNα, IFNλ1, and TNFα production in response to TLR stimulation. This is in line with other studies reporting an impairment of TLR9 expression on blood pDCs associated with an alteration of pDC functions in HBV context (23, 24). Interestingly, in response to TLR triggering, intrahepatic pDCs were also impaired in their maturation capacity. The inability to reveal IFNα production by pDCs following intracellular cytokine staining from whole blood after triggering with classical concentrations of CpG_A_ (not shown) is concordant with others studies (31). However, stimulation of PBMCs with standard concentrations of CpG_A_ showed an impaired production of IFNα and IFNβ from chronic HBV patients, both in proportion and in amount of cytokine per pDCs. Our analysis revealed correlations between pDCs modulations and HBsAg and HBV DNA, suggesting that pDCs status is strongly linked to viral parameters. Results found in HBV patients are concordant with the *in vitro* impact of HBV on “healthy” pDCs showing that HBV actively inhibits pDC function through HBsAg and HBeAg, potentially by binding BDCA2 molecules (21). Taken together, these data demonstrated functional defects of pDCs from chronic HBV patients which might contribute to the failure to properly elicit anti-viral immune responses required for long-term viral control. Therefore, skewing pDCs may be a strategy used by HBV to escape anti-viral immunity.

The scarceness of cDC1 subset in blood and liver renders their functional analysis extremely challenging. A single study described that peripheral cDC1 from chronic HBV patients display an altered maturation capacity and reduced IFNλ1-secretion after TLR3 triggering (13). Unprecedentedly, we demonstrate in chronic HBV patients perturbations of their basal activation status, reduction of OX40L and 4-1BBL molecules expression, and impaired TLR3 sensors associated with an alteration of the maturation capacity and deficient production of IFNλ1, TNFα, and IL-12 following TLRs triggering. Analysis of supernatants of TLR-stimulated PBMCs confirmed the defective IFNλ1 and IFNλ2-secretion following TLR3 triggering. Remarkably, our analyses revealed tight correlations between cDC1 impairment with HBsAg and HBV DNA, suggesting a direct impact of HBV or HBV antigens on cDC1's functionality. This hypothesis is supported by a study showing that, *in vitro*, exposition of “healthy” blood or liver cDC1 to HBsAg altered their capacity to produce IFNλ1 upon TLR3 stimulation (13).

The functional exploration of DCs within liver biopsies is very challenging. In contrast to the reduced IL-12p70, IFNα, IFNβ, IFNλ1, and IFNλ2 cytokine production by PBMCs in response to TLRs stimulation, IL-12, IFNβ, IFNλ1, and IFNλ2 productions per corresponding hepatic DCs were similar in chronic HBV patients vs. controls. Hence, these data suggest that intrahepatic DCs are still functional with a tendency toward an improved cytokine secretion in response to TLR triggering. This is in accordance with a recent study showing that liver specimen from HBV patients did produce comparable IFNs levels as controls (32). These intrahepatic DC features appear tightly linked with HBsAg levels, suggesting a differential impact of HBV within the liver. Indeed, those crucial cytokines, besides being anti-viral, are also pro-inflammatory and can contribute to the liver inflammation during HBV infection (33). Moreover, the higher basal activation status of liver cDC1 followed by similar IFNλs secretion in chronic HBV patients strongly suggest that HBV does not compromise cDC1 ability to promote antiviral immune response. In addition, the fact that intrahepatic cDC1 and pDCs were less activable regarding co-activation markers upon TLR triggering but display similar cytokine secretion, may suggest the existence of distinct functional subtypes of each DC subset as recently proposed (34), reflecting diversity and division of labor between innate and adaptive function of the cells. Moreover, our study revealed increased production of MCP1 from HBV patients after TLRs triggering of PBMCs, suggesting that, HBV might also favor immune cell recruitment at infection sites where they can increase the inflammation process and liver damages as it has been reported (35). Hence, HBV and/or HBV proteins can impact DC's function to favor the chronic inflammation itself. Remarkably, we showed an increased production of the immunosuppressive cytokine TGF-β1 by PBMCs from HBV patients which was maintained upon TLRs stimulation. This is consistent with reported studies showing an up-regulation of TGF-β1 in HBV patients, associated with disease severity (36). In a pioneering way, we highlighted that most of the alterations are subverted on all circulating DCs associated with the potentiation of liver inflammation by intrahepatic DCs. Hence, a subverted DC can potentially, in turn, cross-regulate other immune cell subsets. This hypothesis is supported by some studies presenting a cross-regulation between type I IFN and TNFα in immune-mediated inflammatory disease (37). These data suggest that modulations of circulating and intrahepatic DC features can result from direct impact of HBV, or from interactions of DC components with HBV proteins, as well as from cross-modulations of anterior modulated parameters on others.

Combined, our findings clearly demonstrate that the features of the 3 major DC subsets are deeply subverted in chronic HBV patients in blood but differentially modulated in liver, and highlight the clinical relevance of these observations. As DCs are crucial in driving anti-viral and pro-inflammatory responses, their phenotypic and functional alterations by HBV may subsequently impair proper cross-presentation of viral antigens (38), skew activation of cytotoxic effectors such as T cells and NK cells (25), and prevent elicitation of B cell immunity (39) which is ultimately required to counteract HBV infection; and such DC's hijacking may therefore favor disease persistence. The major collapse of type I and type III IFNs hampered by HBV represent substantial evidences suggesting a deep breakdown of the innate immunity. Together with hijacking of other innate sensors (40), this might alter the induction of effective adaptive immune responses leading to a persistent HBV infection. However, by targeting hepatic cDC2 and pDCs or cDC1 with TLR4, TLR9, and TLR3 ligands, respectively, local production of IL-12 and type I and type III IFNs, which are important for HBV clearance, could be achieved. Thus, our findings bring novel insights into the mechanisms of HBV escape from immune control. They provide bases for designing innovative immunotherapeutic strategies aiming at restoring DC functions through unlocking of the inhibition triggered by the virus, and allowing to restore efficient immune control of the virus and compromise the chronicity of infection.

## Author Contributions

CA, DD, NB-V, VL, and LO: study concept and design; LO and TD-D: acquisition of data; LO, CA, NB-V, DD, VL, TD-D, and LC: analysis and interpretation of data; LO and CA: statistical analyses; VL and TD: material support; MH and JV-G: technical support; LO and CA: drafting of the manuscript; CA, LO, VL, NB-V, LC, DD, and TD: manuscript revision.

### Conflict of Interest Statement

The authors declare that the research was conducted in the absence of any commercial or financial relationships that could be construed as a potential conflict of interest.

## Abbreviations

ALT: alanine aminotransferase
CD: cluster of differentiation
cDC: conventional dendritic cells
CPG_A_: class-A CPG
DC: dendritic cell
DNA: deoxyribonucleic acid
GITRL: glucocorticoid induced TNFR-related protein ligand
HLA-DR: human leukocyte antigen D Related
HBV: hepatitis B virus
HBeAg: hepatitis B e antigen
HBsAg: hepatitis B s antigen
ICOSL: Inducible T-cell co-stimulator ligand
IL-12: interleukin 12
IFN: interferon
LMNCs: liver mononuclear cell
MCP-1: monocyte chemoattractant protein 1
ns: non-significant
pDC: plasmacytoid DC
NK: natural killer cells
PBMCs: peripheral blood mononuclear cells
PDL1: programmed death ligand 1
polyI:C: polyinosinic:polycytidylic acid
OX40L: OX40 ligand
TNFα: tumor necrosis factor α
TLR: toll like receptor; 4-1BB (CD137)
4-1BBL: 4-1BB ligand.
