# Supplementary material for: Circulating and Hepatic BDCA1+, BDCA2+, and BDCA3+ Dendritic Cells Are Differentially Subverted in Patients With Chronic HBV Infection
Source: Front Immunol. 2019 Feb 4;10:112. doi: 10.3389/fimmu.2019.00112 (PMC6369167; doi:10.3389/fimmu.2019.00112)

frontiers

Supplementary Material

Circulating and hepatic BDCA1+, BDCA2+, and BDCA3+ dendritic cells are differentially subverted in patients with chronic HBV infection

Laurissa Ouaguia [1,2], Vincent Leroy [3,4,5], Tania Dufeu-Duchesne [1,4], David Durantel[6], Thomas Decaens [3,4,5], Margaux Hubert[6], Jenny Valladeau-Guilemond[6], Nathalie Bendriss-Vermare[6], Laurence Chaperot [1,2], Caroline Asspord [1,2,*]

[1]Institute for Advanced Biosciences, Research Center Inserm U1209/CNRS 5309/UGA, Immunobiology and immunotherapy of chronic diseases, Grenoble, France.
[2]EFS Rhone-Alpes Auvergne, R&D Laboratory, La Tronche, F-38701, France.
[3]Université Grenoble Alpes, Grenoble, France.
[4]CHU Grenoble Alpes, Hepato-gastroenterology unit, Grenoble, F-38043, France.
[5]Institute for Advanced Biosciences, Research Center Inserm U1209/CNRS 5309/UGA, Analytic Immunology of chronic pathologies, La Tronche, F-38706, France.
[6]Univ Lyon, Université Claude Bernard Lyon 1, INSERM 1052, CNRS 5286, Centre Léon Bérard, Centre de Recherche en Cancérologie de Lyon, Lyon, 69373, France.

***Correspondence:**
Caroline Asspord**,** EFS - R&D Laboratory, EMR EFS-UGA-INSERM U1209-CNRS, Immunobiology and Immunotherapy of Chronic Diseases, 29 avenue du Maquis du Gresivaudan - 38701 La Tronche ; phone : +33 (0)4 76 42 94 83, email : caroline.asspord@efs.sante.fr

Word count: 2272

Number of figures: 6 Supplementary Tables, 12 Supplementary Figures

1 Supplementary Tables

1.1 **Supplementary Table 1**: Clinical characteristics of patients included in this study.

1.2 **Supplementary Table 2: Correlations between peripheral BDCA1+ cDC2 features and clinical parameters in HBV patients**. The frequency and the expression of co-stimulatory and co-inhibitory as well as the maturation and intracellular cytokine expression upon TLR triggering were evaluated within BDCA1+ cDC2 from fresh PBMCs isolated from HBV patients and correlated with the clinical viral parameters. Spearman's correlations between the BDCA1+ cDC2 features and HBV DNA (n=10-12) and/or plasmatic HBsAg levels (n=23-30). Only spearman r≥0.5 or r≤-0.5 with P≤.05 and 0.45≤ r ≤0.5 with P≤.01 or -0.5≤ r ≤-0.45 with P≤0.01 are shown. Blue: negative correlation. *P≤.05, **P<.01.

1.3 **Supplementary Table 3: Correlations between peripheral BDCA2+ pDCs features and clinical patterns in HBV patients**. The expression of co-stimulatory, co-inhibitory and TLR molecules as well as the intracellular cytokine expression upon TLRL triggering were evaluated within BDCA2+ pDCs from fresh PBMCs isolated from HBV patients and correlated with the clinical parameters. Spearman's correlations between the BDCA2+ pDCs features and HBV DNA (n=10-12) and/or plasmatic HBsAg levels (n=23-30). Only spearman r≥0.5 or r≤-0.5 with P≤.05 and 0.45≤ r ≤0.5 with P≤.01 or -0.5≤ r ≤-0.45 with P≤.01 are shown. Blue: negative correlation, *P≤.05.

1.4 **Supplementary Table 4: Correlations between peripheral BDCA3+ cDC1 features and clinical patterns in HBV patients.** The expression of co-stimulatory, co-inhibitory and TLR molecules as well as the maturation and intracellular cytokine expression upon TLRL triggering were evaluated within BDCA3+ cDC1 from fresh PBMCs isolated from HBV patients and correlated with clinical parameters. Spearman's correlations between the secreted cytokines with HBV DNA (n=10-12) and plasmatic HBsAg levels (n=22-30). Only spearman r≥0.5 or r≤-0.5 with P≤.05 and 0.45≤ r ≤0.5 with P≤.01 or -0.5≤ r ≤-0.45 with P≤.01 are shown. Blue: negative correlation, Red: positive correlation. *P≤.05, **P<.01.

1.5 **Supplementary Table 5: Correlations between circulating DC subset features and clinical patterns in HBV patients**. PBMCs from HBV patients were cultured for 22hours with or without TLRLs i.e. polyI:C, R848, CPGA (ODN2336) alone or mixed together (MIX: polyI:C+R848+CPGA) and supernatants were harvested for the analysis of secreted cytokines by Luminex technology. IFNα2, IFNλ1 and IFNλ2 secretion by PBMCs were reported to the absolute number of BDCA2+ pDCs and/or BDCA3+ cDC1 present in samples before stimulation with TLRLs by calculating the cytokine production per DC subset in each sample (amount of cytokine / 10^5 DCs). Only spearman r≥0.5 or r≤-0.5 with P≤.05 and 0.45≤ r ≤0.5 with P≤.01 or -0.5≤ r ≤-0.45 with P≤.01 are shown. Blue: negative correlation, *P≤.05, ***P<.001.

1.6 Supplementary Table 6: Correlations between intrahepatic DC subset features and clinical patterns in HBV patients. DC subset's frequencies and expression of co-activation molecules were analyzed by flow cytometry on liver biopsies from chronic HBV patients. The activation status of DC subsets and cytokine secretion by LMNCs were evaluated after TLR triggering with a mixture of TLRL (MIX) and correlated with the clinical parameters. Spearman's correlations between the BDCA1+ cDC2, BDCA2+ pDCs, and BDCA3+ cDC1

features and HBV DNA and plasmatic HBsAg levels (n=10-12). Only spearman r≥0.5 or r≤-0.5 with P≤.05 and 0.45≤ r ≤0.5 with P≤.01 or -0.5≤ r ≤-0.45 with P≤.01 are shown. Blue: negative correlation, Red: positive correlation. *P≤.05, **P<.01.**Supplementary Figures**

2 Supplementary Figures

2.1 Supplementary Figure S1. Circulating and intrahepatic DC subset's absolute numbers and frequencies from chronic HBV patients and healthy donors or non-viral infected controls.

2.1 Supplementary Figure S1. Circulating and intrahepatic DC subset's absolute numbers and frequencies from chronic HBV patients and healthy donors or non-viral infected controls. DC subset's frequencies were analyzed by flow cytometry on peripheral blood and liver biopsies of chronic HBV patients and HD or non-viral infected controls and DC subsets's absolute number calculated. (*A*) DC subsets gating strategy (representative flow cytometry plots of a HBV patient). Fresh cell suspension was analyzed by first gating on lived living cells. The DC populations were identified as CD45+HLA-DR+Lineage- (Lin: CD3/CD14/CD16/CD19/CD20/CD56) mononuclear cells and subdivided as CD11c+CD1c/BDCA1+ cDC2, CD11c-BDCA2+ pDCs, and CD11c+BDCA2-BDCA3+ cDC1. (*B*) DC subsets absolute numbers reported to 1.10^6 PBMCs or LMNCs among alived CD45+ cells. Open symbols, HD or non-viral infected controls (CTRL) (blood, n=22; liver, n=14); filled symbols, chronically HBV-infected patients (HBV) (blood, n=32-33; liver, n=10). *(C)* Spearman's correlations between the frequency of DC subsets from HBV patients and plasmatic HBsAg levels (blood BDCA1+ cDC2, n=31; intrahepatic BDCA2+ pDCs, n = 18). *(D)* Spearman's correlations of frequencies between the three different circulating DC subsets (n=31) or *(E)* intrahepatic DC subsets (n=10) from chronic HBV patients.

2.2 **Supplementary Figure S2. Modulation of the basal activation status of circulating and intrahepatic DCs in chronic HBV patients.** The expression of the co-activation molecules CD40, CD80, and CD86 was analyzed by flow cytometry on peripheral blood and liver biopsies of chronic HBV patients and HD or non-viral infected controls. *(A)* Representative flow cytometry plots of a HD or CTRL and HBV patient. Fresh cell suspension was analyzed by first gating BDCA1+ cDC2, BDCA2+ pDCs, and BDCA3+ cDC1 as described above and the expression of activation molecules (here CD40) on each subset was analyzed. *(B)* MFI level of CD40, CD80, and CD86 on circulating cDC2, pDCs and cDC1. Open symbols, HD (n=17-21); filled symbols, HBV (n=25-31). *(C)* MFI level of CD40, CD80 and CD86 on intrahepatic DC subsets. Open symbols, CTRL (n=11-15); filled symbols, HBV (n =8-12). Bars indicate mean. *P*-values were calculated using the 2-way-RM ANOVA test (straight line) and Mann–Whitney test (dashed lines). *(D)* Spearman's correlations of CD40 MFI on intrahepatic BDCA3+ cDC1 from HBV patients with plasmatic HBsAg levels (n=12). *(E)* Spearman's correlation of CD80 expression between peripheral BDCA1+ mDC and BDCA3+ mDC subsets in chronic HBV patients (n=30). *(F)* Spearman's correlation of %CD80 between intrahepatic BDCA1+ cDC2, BDCA2+ pDCs, and BDCA3+ cDC1 from chronic HBV patients (n=11-12).

2.3 **Supplementary Figure S3. Modulation of co-stimulatory molecules expression on circulating BDCA2+ pDCs in chronic HBV patients.** OX40L, 4-1BBL and ICOSL expressions were determined by flow cytometry on peripheral DC subsets from fresh PBMCs isolated from HD and HBV patients. *(A)* Representative flow cytometry plots of a HD and HBV patient. Fresh cell suspension was analyzed by first gating BDCA1+ cDC2, BDCA2+ pDCs, and BDCA3+ cDC1 as described above and the expression of activation molecules (here OX40L) on each subset was analyzed. *(B)* MFI level of OX40L, 41-BBL, GITRL and

ICOSL on circulating cDC2, pDCs and cDC1. Open symbols, HD (n=17-18); filled symbols, HBV (n=21-26). *(C)* Flow cytometry quantification of PDL1 expression on BDCA1+ cDC2, BDCA2+ pDCs and BDCA3+ cDC1 from fresh PBMCs of HBV patients or HD. Open symbols, HD (n=21); filled symbols, HBV patients (n =29-31). Bars indicate mean. *P*-values were calculated using the 2-way-RM ANOVA test (straight line). *(D)* Correlations between %4-1BBL on circulating BDCA3+ cDC1 from viremic HBV patients with HBV DNA (n=11) (Spearman correlation).

2.4 **Supplementary Figure S4. TLR expression among circulating DC subsets.** Fresh cell suspensions were analyzed by first gating BDCA1+ cDC2, BDCA2+ pDCs, and BDCA3+ cDC1 as described above and TLR expression was analyzed in corresponding DC subset. *(A)* Gating strategy. Surface TLR4 and intracellular TLR8 in BDCA1+ cDC2, intracellular TLR9 in BDCA2+ pDCs; and intracellular TLR3 and TLR8 in BDCA3+ cDC1. Representative flow cytometry plots of a HD and HBV patient. *(B)* MFI level of TLR8 and TLR4 on cDC2; TLR9 on pDCs; and TLR3 and TLR8 on cDC1. Open symbols, HD (n=14-15 for TLR3,8,9 and n=24 for TLR4); filled symbols, patients with chronic HBV (n=22-24 for TLR3,7,9 and n=28-35 for TLR4). Bars indicate median. *P*-values were calculated using the Mann-Whitney test.

2.5 Supplementary Figure S5. Altered maturation of blood DC subsets in chronic HBV patients upon TLRL stimulation. PBMCs from HD or HBV patients were cultured for 22hours with or without polyI:C, R848, CPG_A (ODN2336) alone or mixed together (MIX: polyI:C+R848+CPG_A). Flow cytometry quantification of CD40, CD80, and CD86 (MFI) on positive cells within *(A)* BDCA1+ cDC2, *(B)* BDCA2+ pDCs, and (C) BDCA3+ cDC1. Open symbols, HD (n=13-18); filled symbols, HBV patients (n=22-26). *P*-values were calculated using the 2-way-ANOVA test (straight line, **P*<.05, ***P*<.01) or the Mann–Whitney test (dashed lines).

2.6 Supplementary Figure S6. Correlations of maturation molecules modulation upon TLR triggering between circulating DC subsets in chronic HBV patients. PBMCs from chronic HBV patients were cultured for 22hours with or without polyI:C, R848, CPG_A (ODN2336) alone or mixed together (MIX: polyI:C+R848+CPGA). The expression of the maturation markers CD40, CD80, and CD86 was measured on blood DC subsets by flow cytometry. *(A)* Spearman’s correlations of single maturation marker modulation after TLR stimulation between peripheral BDCA1+ cDC2, and BDCA2+ pDCs; *(B)* Spearman’s cross-correlations between distinct maturation markers expression under TLR stimulation between peripheral BDCA1+ cDC2 and BDCA2+ pDCs in chronic HBV patients (Spearman correlation, n=23-24). Only spearman r≥0.5 or r≤-0.5 with *P*≤.05 and r≥0.45 or r≤-0.45 with *P*≤.01 are shown.

2.7 Supplementary Figure S7. Correlations of maturation molecules modulation upon TLR triggering between intrahepatic DC subsets in chronic HBV patients. LMNCs from chronic HBV patients were cultured for 22hours with or without mixed TLRL (MIX: polyI:C+R848+CPGA). The expression of the maturation markers CD40, CD80 and CD86

was measured on hepatic DC subsets by flow cytometry. Spearman's correlations of CD86 expression after stimulation with mixed TLRL between intrahepatic *(A)* BDCA1+ cDC2 and BDCA2+ pDCs, *(B)* BDCA1+ cDC2 and BDCA3+ cDC1, and *(C)* BDCA3+ cDC1 and BDCA2+ pDCs in chronic HBV patients (n=10-11).

2.8 Supplementary Figure S8. Correlations between the intracellular productions of antiviral cytokines by circulating DC subsets in chronic HBV patients after TLRLs triggering. Whole blood samples were cultured for 5hours with or without polyI:C, R848, CPG_A (ODN2336) alone or mixed together (MIX: polyI:C+R848+CPG_A) and the production of intracellular cytokines by each subset was measured by flow cytometry. *(A)* Representative flow cytometry plots of a HD and HBV patient. Fresh cell suspension was analyzed by first gating BDCA1+ cDC2, BDCA2+ pDCs, and BDCA3+ cDC1 as described above and the intracellular production of corresponding cytokines (here IL12p40/70 for BDCA1+ cDC2, IFNα for BDCA2+pDCs, and IFNλ1 for BDCA3+ cDC1) on each subset was analyzed. Spearman correlations between the intracellular cytokine production by peripheral DC subsets from HBV patients within *(B)* IL-12p40/70 and TNFα by BDCA1+ cDC2, *(C)* TFNα and IFNα by BDCA2+ pDCs and *(D)* TNFα, IL-12p40/70, and IFNλ1 by BDCA3+ cDC1 (n=23). Only spearman r≥0.5 or r≤-0.5 with $P \leq .05$ and r≥0.45 or r≤-0.45 with $P \leq .01$ are shown.

2.9 Supplementary Figure S9. The impaired productions of intracellular IL-12p70, TNFα, IFNα and IFNλ1 (IL-29) cytokines after TLRLs triggering are tightly correlated among peripheral DC subsets in chronic HBV patients. Whole blood samples were cultured for 5hours with or without polyI:C, R848, CPG_A (ODN2336) alone or mixed together (MIX:polyI:C+R848+CPGA) and the production of intracellular cytokines by each subset was measured by flow cytometry. Cross-correlations between the intracellular cytokine production were performed between *(A)* IL-12p70/TNFα-producing BDCA1+ cDC2 and IFNα/TNFα-producing BDCA2+ pDCs, *(B)* IL-12p70-producing BDCA1+ cDC2 and IFNλ1-producing BDCA3+ cDC1, and *(C)* IFNα-producing BDCA2+ pDCs and IFNλ1-producing BDCA3+ cDC1 (spearman correlation, n=24). *(D)* Spearman correlations between cytokine production by corresponding blood BDCA2+ pDCs and BDCA3+ cDC1 with HBsAg levels (n=18-20) and viral DNA (n=9). Only spearman r≥0.5 or r≤-0.5 with $P \leq .05$ and r≥0.45 or r≤-0.45 or r≥-0.45 with $P \leq .001$ are shown.

2.10 Supplementary Figure S10. Hepatitis B infection impairs IFNα2, IFNß, IFNλ1 and IFNλ2 anti-viral cytokine secretion from chronic HBV patients after TLR triggering. DC subset's frequencies were analyzed by flow cytometry on PBMCs of chronic HBV patients and HD as previously described. PBMCs of HD or HBV patients were cultured for 22hours with or without polyI:C, R848, CPG_A (ODN2336) alone or mixed together (MIX:polyI:C+R848+CPG_A) and culture supernatants were examined for the secretion of IL-12p70, IFNα2, IFNß, IFNλ1 (IL-29), IFNλ2 (IL-28A), TNFα, and TGFß1 by Luminex technology. *(A)* Comparative cytokine secretion in HD and HBV patients (pg/ml). Open symbols, HD (n=14-16); filled symbols, HBV patients (n=26-27). *(B)* IFNα2, IFNλ1 and IFNλ2 secretions by PBMC suspensions were reported to the absolute number of BDCA2+

pDCs and/or BDCA3+ cDC1 present in samples before stimulation and correlated with the viral DNA (n=11-12) in HBV patients.

2.11 Supplementary Figure S11. Modulation of the secretion of MCP-1 chemokine and increased regulatory TGF-ß1 secretion from PBMCs in HBV context after TLRLs triggering. PBMCs from chronic HBV patients and HD were cultured for 22hours with or without polyI:C, R848, CPG_A (ODN2336) alone or mixed together (MIX:polyI:C+R848+CPG_A) and the supernatant of culture was examined for cytokine secretion by Luminex technology. *(A)* Comparative secretion of MCP-1, IP-10, IL-10 and TGFß1 in HD and HBV patients (pg/ml). Open symbols, HD (n=14-16); filled symbols, patients with chronic HBV (n=27-28). *P*-values were calculated using the 2-way-RM ANOVA test (*P≤.05, straight line) and the Mann–Whitney test (dashed lines). *(B)*Spearman's correlations between MCP-1 cytokine secretion by PBMCs from HBV patients and plasmatic HBsAg levels (n=27).

2.12 Supplementary Figure S12. Intrahepatic cytokine secretion. Liver-infiltrating cell suspensions ($1X10^6$cells/ml) from HBV patients were stimulated for 22hours with or without a mixture of TLRL (MIX:polyI:C+R848+CPG_A) and the supernatant of the cultures supernatants were examined for the presence of IL-12p70, IFNβ, IFNλ1, IFNλ2, TNFα, IP10, MCP1, IL-10, and TGF-ß1 by Luminex technology. *(A)* Table summarizing the quantification of cytokine secretions in the supernatants from HBV biopsy samples (n=2) in absence of stimulation (media) or with a mixture of TLRL (MIX). *(B)* Correlations between the secretions of IL-12p70, TNFα, IFNβ, IFNλ1 (IL-29) and IFNλ2 (IL-28A) cytokines after TLRLs triggering of LMNCs in chronic HBV patients. DC subset's frequencies were analyzed by flow cytometry on LMNCs of chronic HBV patients as previously described. Cytokine's secretion by liver cell suspensions were reported to the absolute number of DCs present in samples before stimulation with the mixture of TLRLs. Correlations between IL-12p70 reported to cDC1and cDC2; and IFNλ2 reported to cDC1 and pDCs; and IFNβ reported to pDCs and IFNλ1 or IFNλ2 reported to cDC1and pDCs (spearman correlation, n=12).

Supporting Table 1

	Blood samples						Liver biopsy samples	
**Classification**	HBeAg positive			HBeAg negative			Chronic Hepatitis B	Non viral liver diseases[f]
	Chronic infection	Chronic hepatitis		Chronic infection	Chronic hepatitis			
**Old terminology**	Immune tolerant	Immune reactive		Inactive carriers	HBeAg negative chronic hepatitis			
		Untreated	Treated		Untreated	Treated	Untreated	
**n**	3	4	10	39	10	64	29	33
**Age (y)**	27 ± 9.8	35 ± 11.3	43 ± 18.4	39 ± 14.6	34 ± 14.7	54±15.8	37 ± 14.2	55 ± 11.2
**Sex (%, Male/Female)**	66.7/33.3	100/0	90/10	74.3/25.7	60/40	76.6/23.4	69/31	51.5/48.5
**ALT level (IU/L)**	76.3 ± 54.3	59 ± 49.2	48 ± 15.7	38 ± 16.6	54 ± 40.1	36 ± 14.2	62 ± 39.9	72 ± 42.3
**HBV DNA (log10 IU/mL)**	8.2 ± 0	6.7 ± 0.6	1.2 ± 0.1	2.4 ±0.7	3.9 ± 0.4	1.2 ± 0.2	4.4 ± 1.9	NA
**HBsAg (log10 IU/mL)**	4.6± 0.02	3.9 ± 0.6	3.3 ± 1.1	3.4 ± 0.99	3.8 ± 0.4	3.1± 0.99	3.8 ± 0.5	NA
**On treatment (%)**	0[a]	0[a]	100[b]	0[a]	0[a]	100[c]	3.4[d]	NA
**METAVIR A**	ND	2 ± 0.7[e]	2 ± 0.9[e]	1 ± 0.7[e]	ND	1± 0.8[e]	1 ± 0.8	2 ± 1.2
**METAVIR F**	ND	1 ± 0[e]	1 ± 0.98[e]	1 ± 1.05[e]	ND	2±1.04[e]	1 ± 1.003	1 ± 1.2
**F3F4 (%)**	ND	0[e]	12.5[e]	8.3[e]	ND	18.9[e]	13,8	10.7

Data are expressed as mean ± SD unless indicated otherwise

b) No treatment (100%)

b) Tenofovir (100%)

c) 3,12% Entecavir+Tenofovir, 68,75% Tenofovir, 21,86% Entecavir, 4,69% Lamavudine, 1,56% Adefovir

d) 96.55% No Treatment (28),3.44% Entecavir (1)

e) Values calculated on patients with known METAVIR A and F Score

f) 28 nonalcoholic metabolic steatohepatitis, 2 unknown hypertransaminasemia, 1 primary sclerosing cholangitis, 1 primary biliary cholangitis and 1 unknown steatopathy

Abreviations: ALT, alanine aminotransferase; F, female; HBeAg, hepatitis B e antigen; HBsAg, hepatitis B s antigen; HBV, hepatitis B virus; M, male ; n, number of patient; NA, not applicable; ND, not determined; y, year; %, percentage

Supporting Table 2

Circulating BDCA1+ cDC2			Viral patterns	
		TLRL stimulation	HBsAg IU/mL	HBV DNA IU/mL on viremic HBV patients
Frequency within CD45+	% Frequency	-	**	
Immune checkpoints: Costimulatory and coinhibitory molecules	% OX40L+		*	
	%PDL1+			*
Maturation after TLRs triggering	%CD40+	polyI:C	*	
		MIX		*
	%CD80+	polyI:C	*	
	MFI CD80+	MIX		*
	%CD86+	polyI:C	**	
		MIX	*	
Intracellular cytokine expression after TLRs stimulation	%IL-12p40/70+	MIX	*	

Spearman correlation r≥0.5 or r≤- 0.5 with *P* ≤.05

and 0.45≤ r ≤0.5 with *P* ≤.01

or -0.5≤ r ≤-0.45 with *P* ≤.01

Blue : negative correlation

*P ≤.05, **P <.01

Supporting Table 3

Circulating BDCA2+ pDCs			Viral patterns	
		TLRL stimulation	HBsAg IU/mL	HBV DNA IU/mL on viremic HBV patients
**Immune checkpoints: Costimulatory and coinhibitory molecules**	% OX40L+	-		*
	%PDL1+			*
**Intracellular cytokine expression after TLRs stimulation**	% IFNα	MIX	*	
	%IFNλ1	R848		*
		MIX		*
	%TNFα+	R848	*	

Spearman correlation r≥0.5 or r≤- 0.5 with *P* ≤.05

and 0.45≤ r ≤0.5 with *P* ≤.01

or -0.5≤ r ≤-0.45 with *P* ≤.01

Blue : negative correlation

*P ≤.05

Supporting Table 4

Circulating BDCA3+ cDC1			Viral patterns	
		TLRL stimulation	HBsAg IU/mL	HBV DNA IU/mL on viremic HBV patients
Immune checkpoints: Costimulatory and coinhibitory molecules	%41BBL+	-		*
Maturation after TLRLs	MFI CD40+	CPGA	**	
	% CD80	polyI:C	*	
	MFI CD80+	R848	**	
		CPGA	**	
	%CD86+	R848		*
	MFI CD86+	polyI:C	*	
		MIX	*	
Intracellular cytokine expression after TLRLs stimulation	%IFNλ1+	polyI:C		*
		R848		*

Spearman correlation r≥0.5 or r≤- 0.5 with *P* ≤.05

and 0.45≤ r ≤0.5 with *P* ≤.01

or -0.5≤ r ≤-0.45 with *P* ≤.01

Blue : negative correlation

Red : positive correlation

**P* ≤.05, ***P*<.01

Supporting Table 5

Circulating DC subsets			Viral patterns
		**TLRL stimulation**	**HBV DNA IU/mL on viremic HBV patients**
**Cytokines per 10^5 BDCA2+ pDC subsets**	IFNα	MIX	***
**Cytokines per 10^5 cDC1 and pDC subsets**	IFNλ1	R848	*
		MIX	*
	IFNλ2	poly I:C	*

Spearman correlation r≥0.5 or r≤- 0.5 with *P* ≤.05

and 0.45≤ r ≤0.5 with *P* ≤.01

or -0.5≤ r ≤-0.45 with *P* ≤.01

Blue : negative correlation

**P* ≤.05, ****P* <.001

Supporting Table 6

<table>
<tr><th colspan="3" bgcolor="#ddd8c4">Intrahepatic DC subsets</th><th colspan="2">Viral patterns</th></tr>
<tr><th colspan="2">Co-Activation molecules and Frequency</th><th>TLRL stimulation</th><th>HBsAg IU/mL</th><th>HBV DNA IU/mL on viremic HBV patients</th></tr>
<tr><td>BDCA1+ cDC2</td><td>% CD40+</td><td>MIX</td><td bgcolor="#1589ec">*</td><td></td></tr>
<tr><td rowspan="2">BDCA2+ pDCs</td><td>% within CD45+</td><td rowspan="2">-</td><td bgcolor="#ff5555">*</td><td></td></tr>
<tr><td>% CD40+</td><td bgcolor="#1589ec">**</td><td bgcolor="#1589ec">*</td></tr>
<tr><td>BDCA3+ cDC1</td><td>% CD80+</td><td>MIX</td><td></td><td bgcolor="#1589ec">*</td></tr>
<tr><th colspan="2">Secreted cytokines</th><td colspan="3"></td></tr>
<tr><td rowspan="4">Cytokines secretion from LMNC</td><td>TGF-β1</td><td>MIX</td><td bgcolor="#ff5555">**</td><td></td></tr>
<tr><td>MCP1</td><td>MIX</td><td bgcolor="#ff5555">*</td><td></td></tr>
<tr><td>IP10</td><td>MIX</td><td bgcolor="#ff5555">*</td><td></td></tr>
<tr><td>IL-12p70</td><td>MIX</td><td bgcolor="#ff5555">*</td><td></td></tr>
</table>

Spearman correlation r≥0.5 or r≤- 0.5 with *P* ≤.05

and 0.45≤ r ≤0.5 with *P* ≤.01

or -0.5≤ r ≤-0.45 with *P* ≤.01

Red : positive correlation

Blue : negative correlation

**P* ≤.05, ***P* <.01

Supporting Figure S1

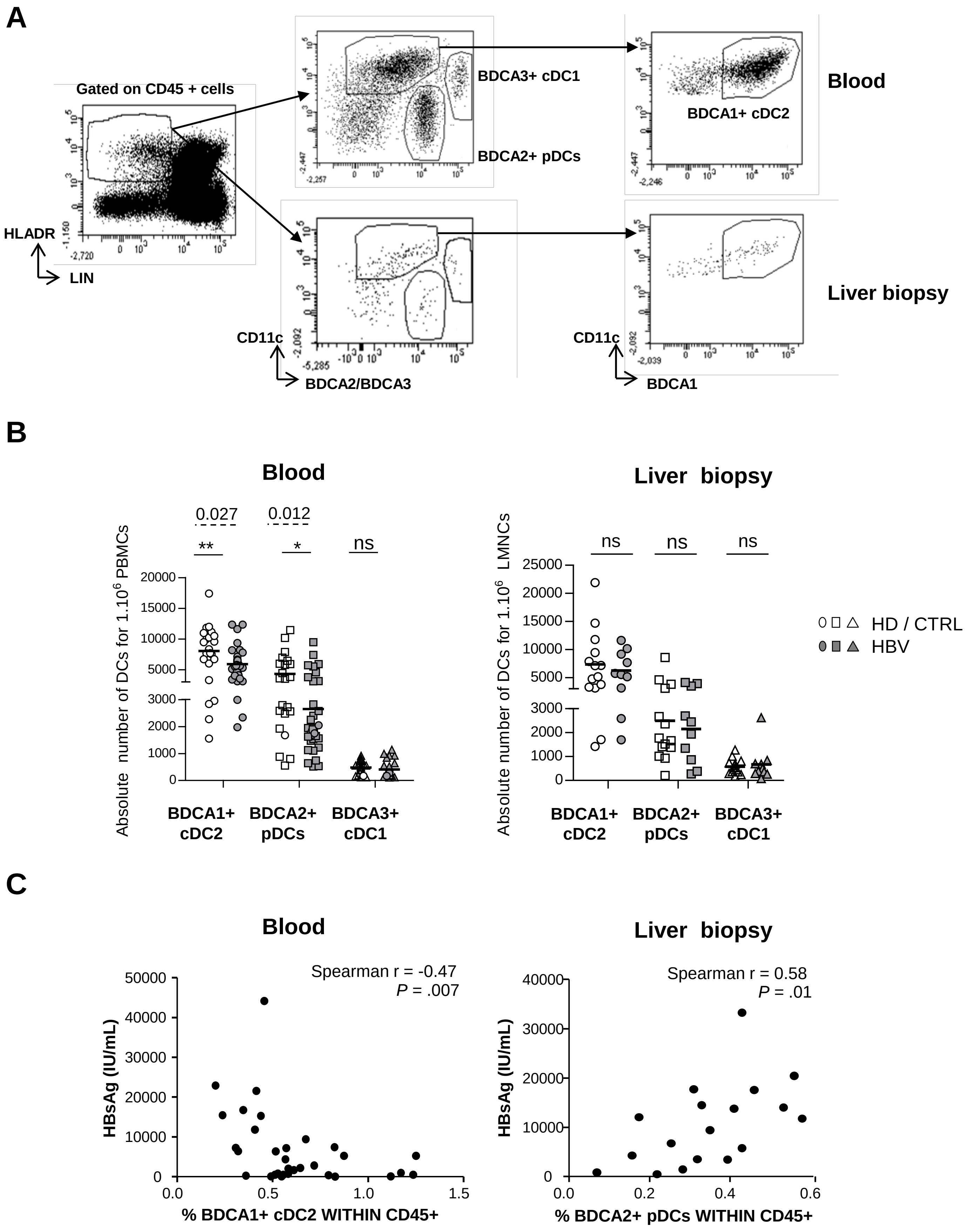

D

Blood

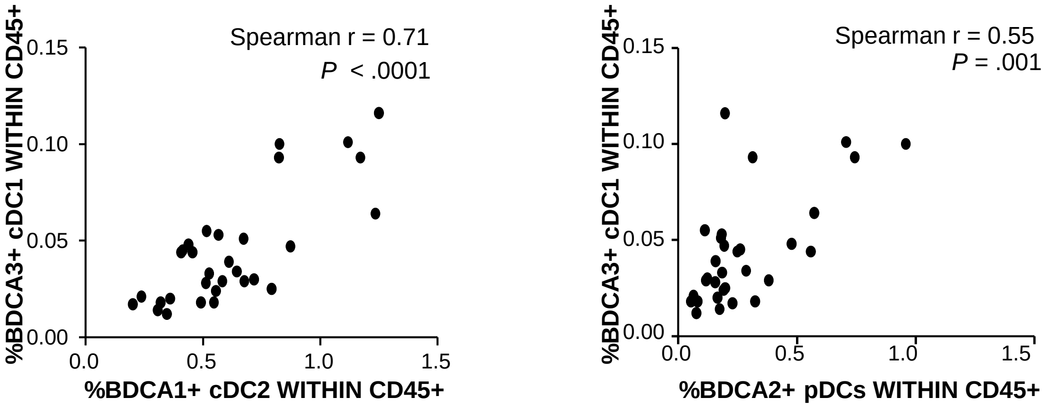

E

Liver biopsy

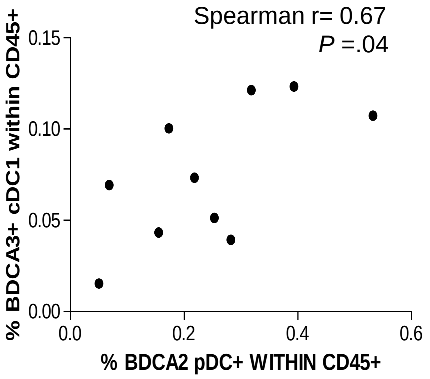

Supporting Figure S2

A

Blood

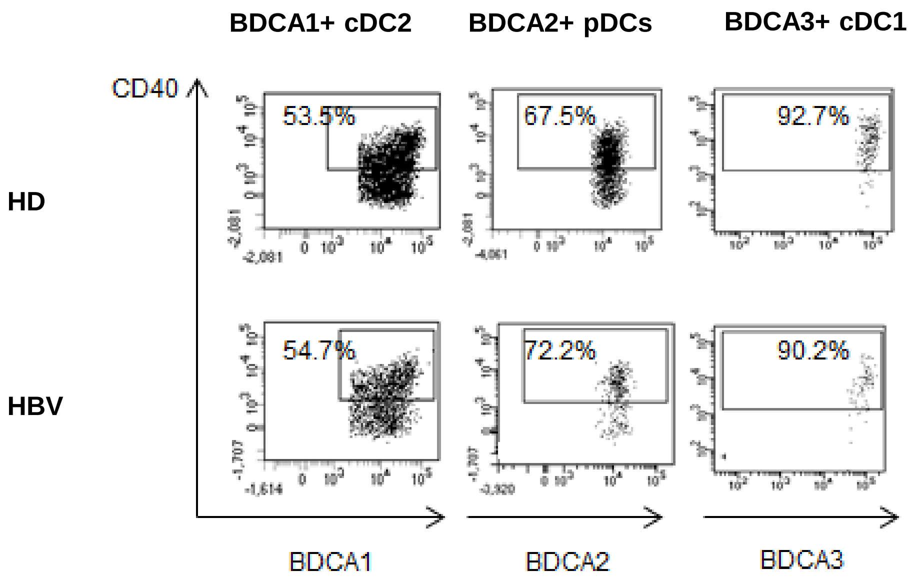

Liver biopsy

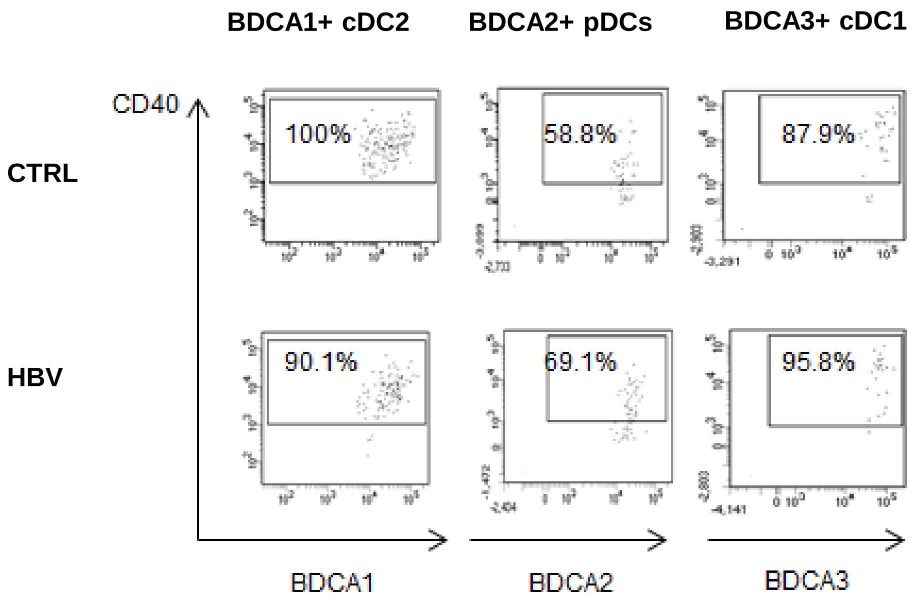

B

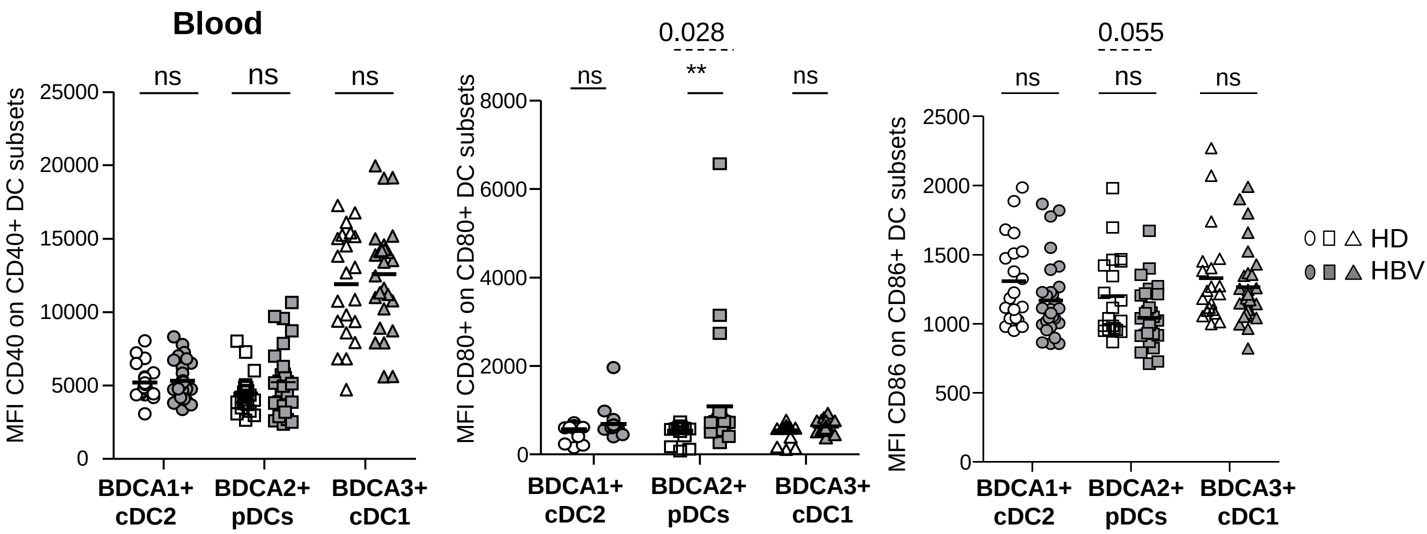
Blood
ns
ns
ns
MFI CD40 on CD40+ DC subsets
25000
20000
15000
10000
5000
0
BDCA1+ cDC2
BDCA2+ pDCs
BDCA3+ cDC1
0.028
ns
**
ns
MFI CD80+ on CD80+ DC subsets
8000
6000
4000
2000
0
0.055
ns
ns
ns
MFI CD86 on CD86+ DC subsets
2500
2000
1500
1000
500
0
HD
HBV

C

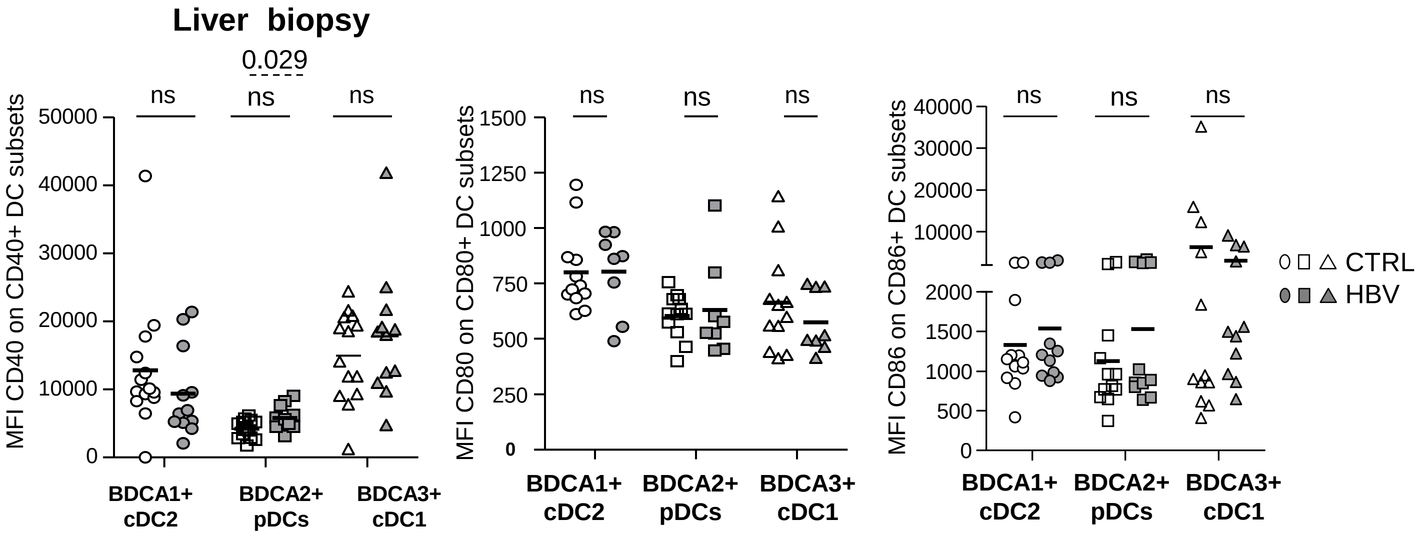
Liver biopsy
0.029
ns
ns
ns
MFI CD40 on CD40+ DC subsets
50000
40000
30000
20000
10000
0
BDCA1+ cDC2
BDCA2+ pDCs
BDCA3+ cDC1
ns
ns
ns
MFI CD80 on CD80+ DC subsets
1500
1250
1000
750
500
250
0
ns
ns
ns
MFI CD86 on CD86+ DC subsets
40000
30000
20000
10000
2000
1500
1000
500
0
CTRL
HBV

D

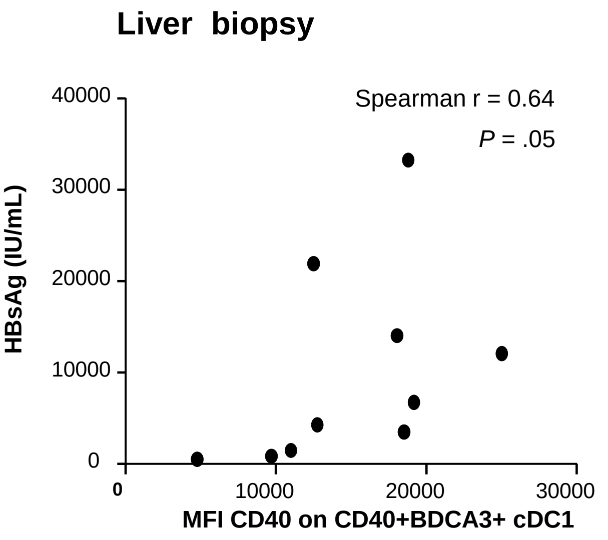
Liver biopsy
Spearman r = 0.64
P = .05
HBsAg (IU/mL)
40000
30000
20000
10000
0
0
10000
20000
30000
MFI CD40 on CD40+BDCA3+ cDC1

E

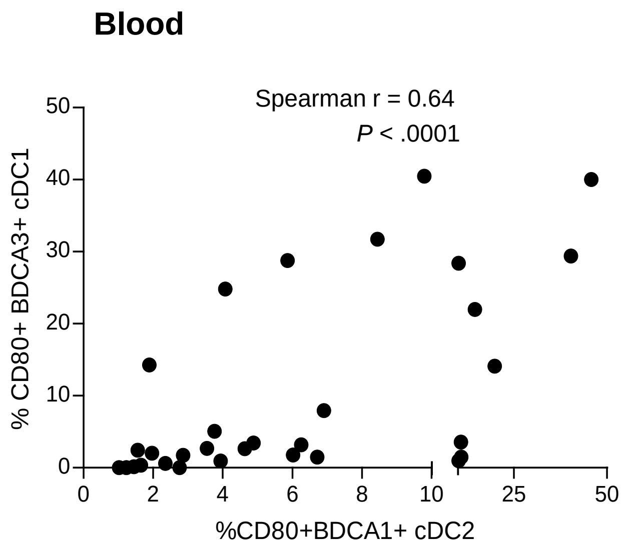
Blood
Spearman r = 0.64
P < .0001
% CD80+ BDCA3+ cDC1
50
40
30
20
10
0
0
2
4
6
8
10
25
50
%CD80+BDCA1+ cDC2

F

Liver biopsy

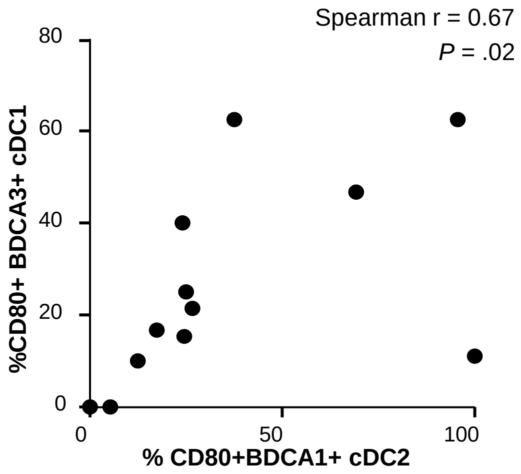

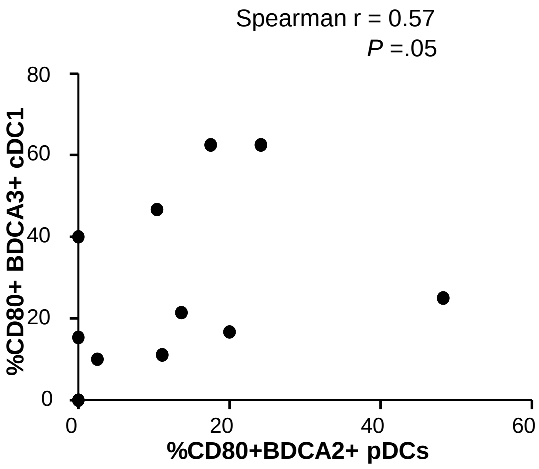

Supporting Figure S3

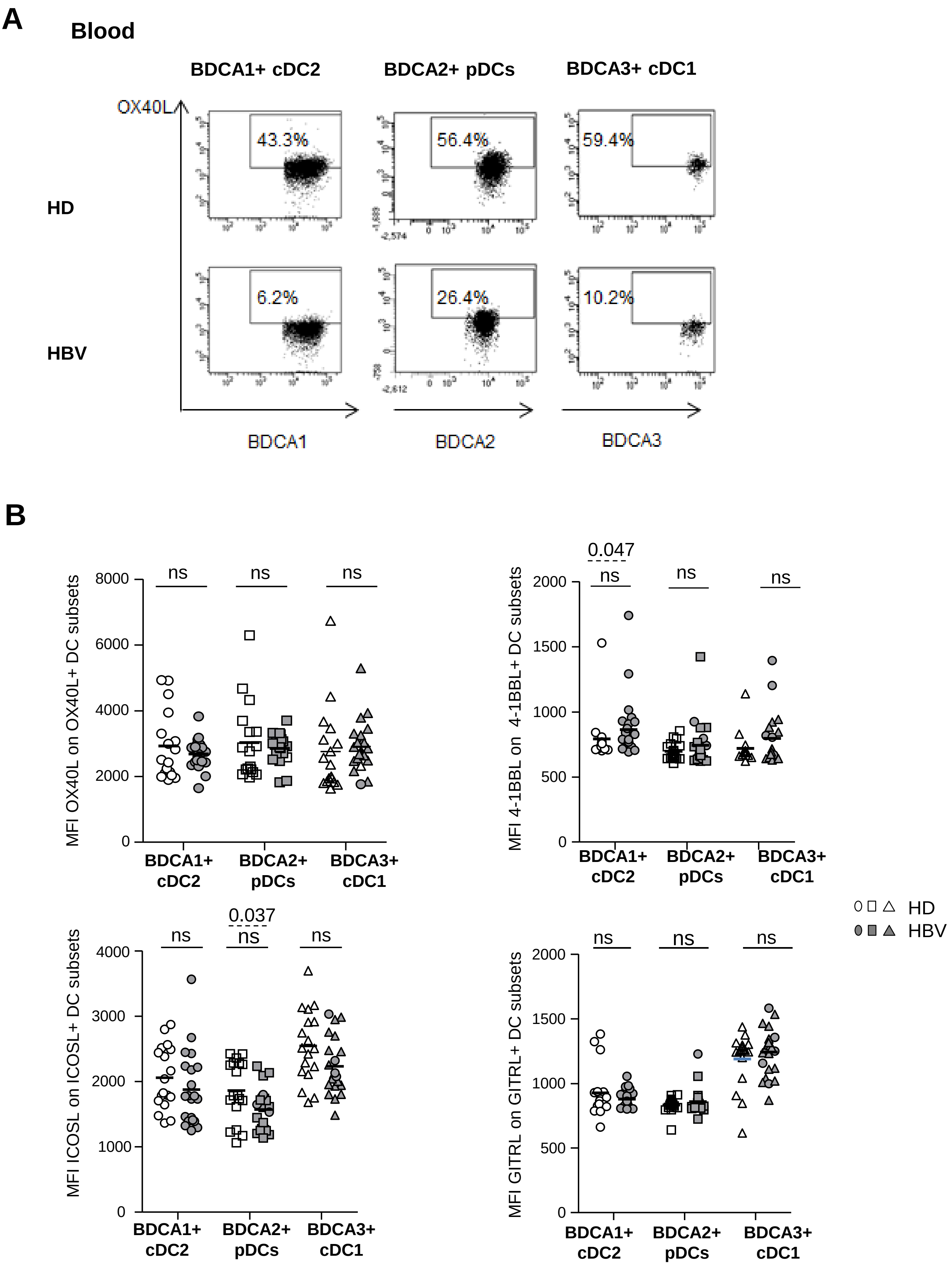

C

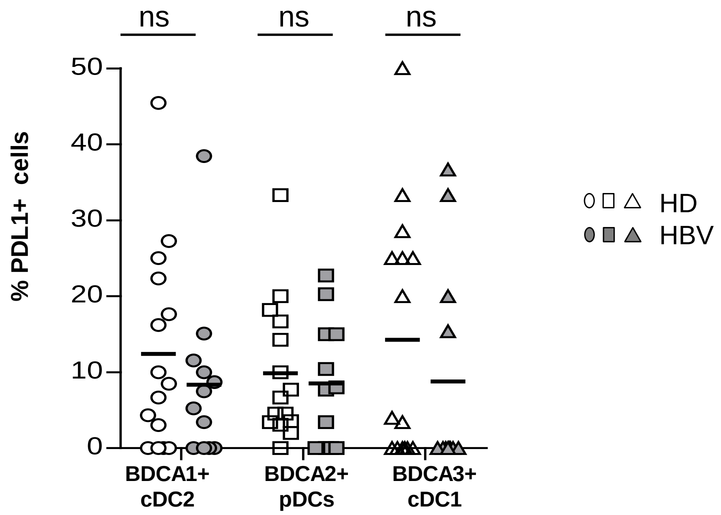
ns
ns
ns
% PDL1+ cells
50
40
30
20
10
0
BDCA1+ cDC2
BDCA2+ pDCs
BDCA3+ cDC1
HD
HBV

D

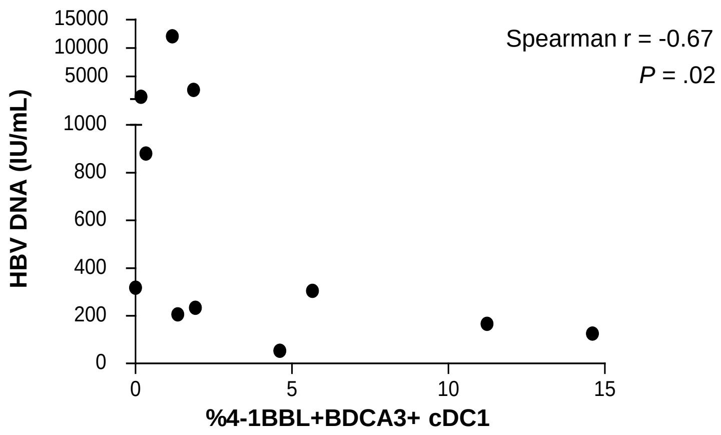
Spearman r = -0.67
P = .02
HBV DNA (IU/mL)
15000
10000
5000
1000
800
600
400
200
0
0
5
10
15
%4-1BBL+BDCA3+ cDC1

Supporting Figure S4

A

Blood

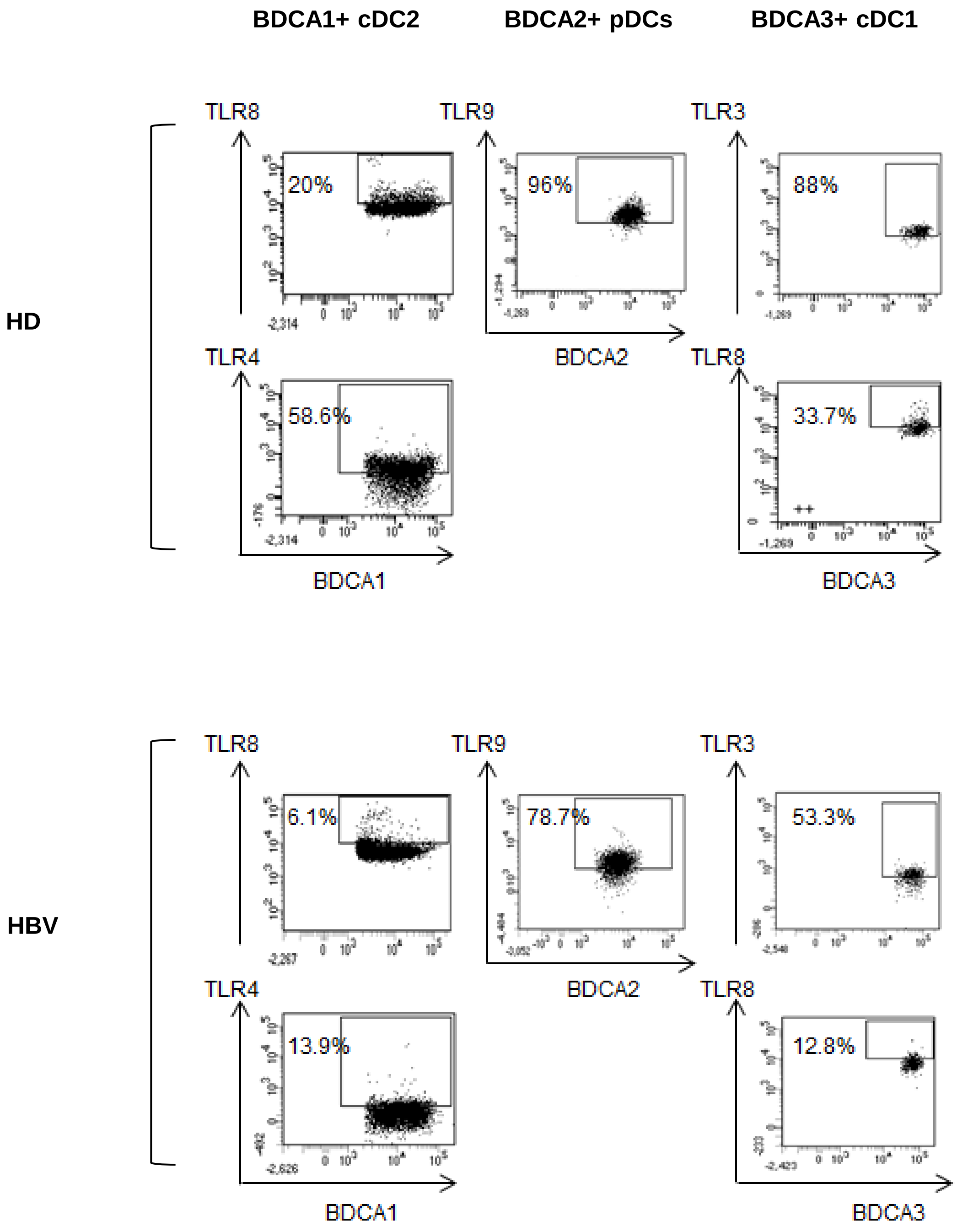

B

Blood

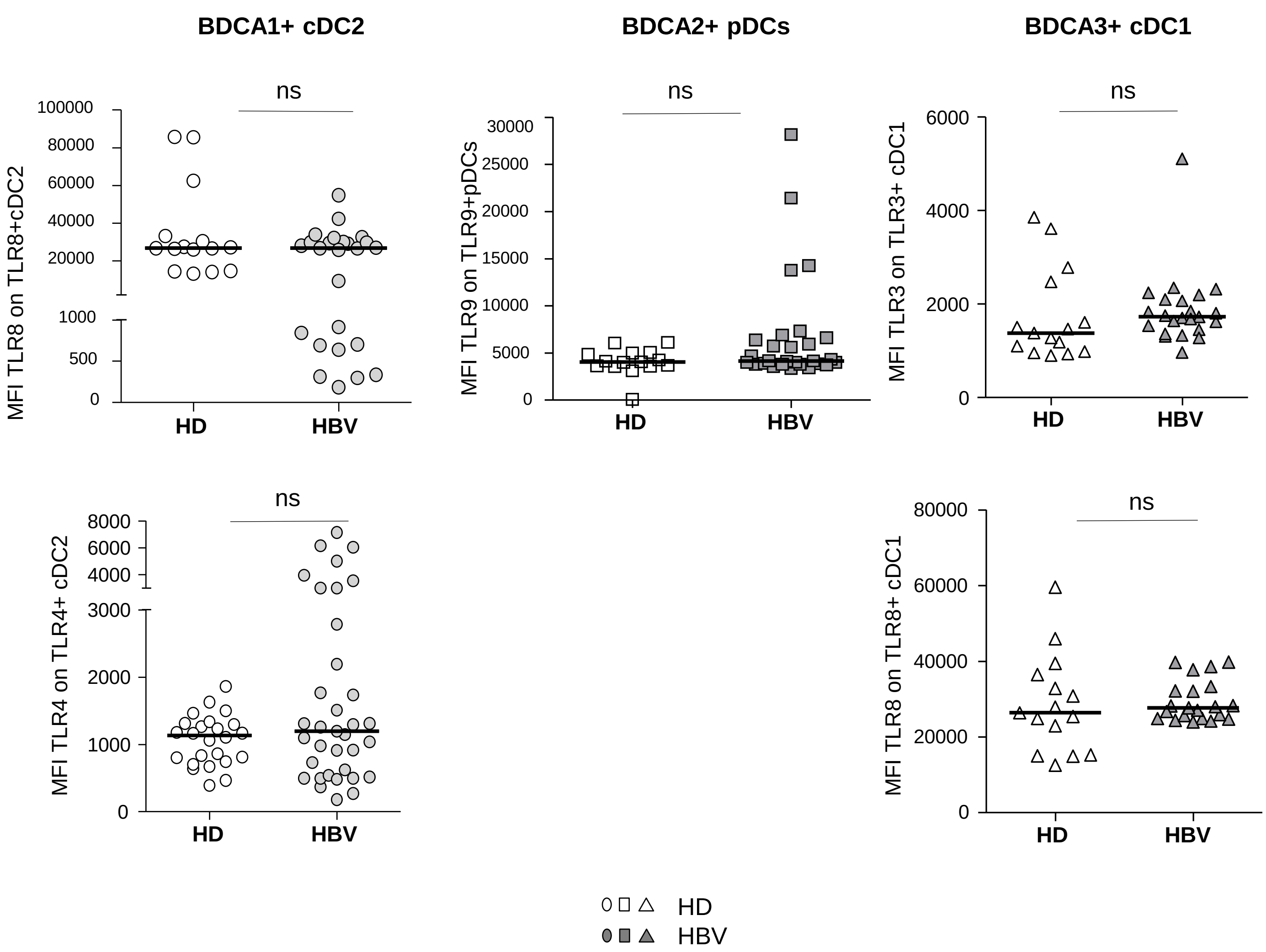

BDCA1+ cDC2
BDCA2+ pDCs
BDCA3+ cDC1
ns
MFI TLR8 on TLR8+cDC2
MFI TLR9 on TLR9+pDCs
MFI TLR3 on TLR3+ cDC1
MFI TLR4 on TLR4+ cDC2
MFI TLR8 on TLR8+ cDC1
HD
HBV

Supporting Figure S5

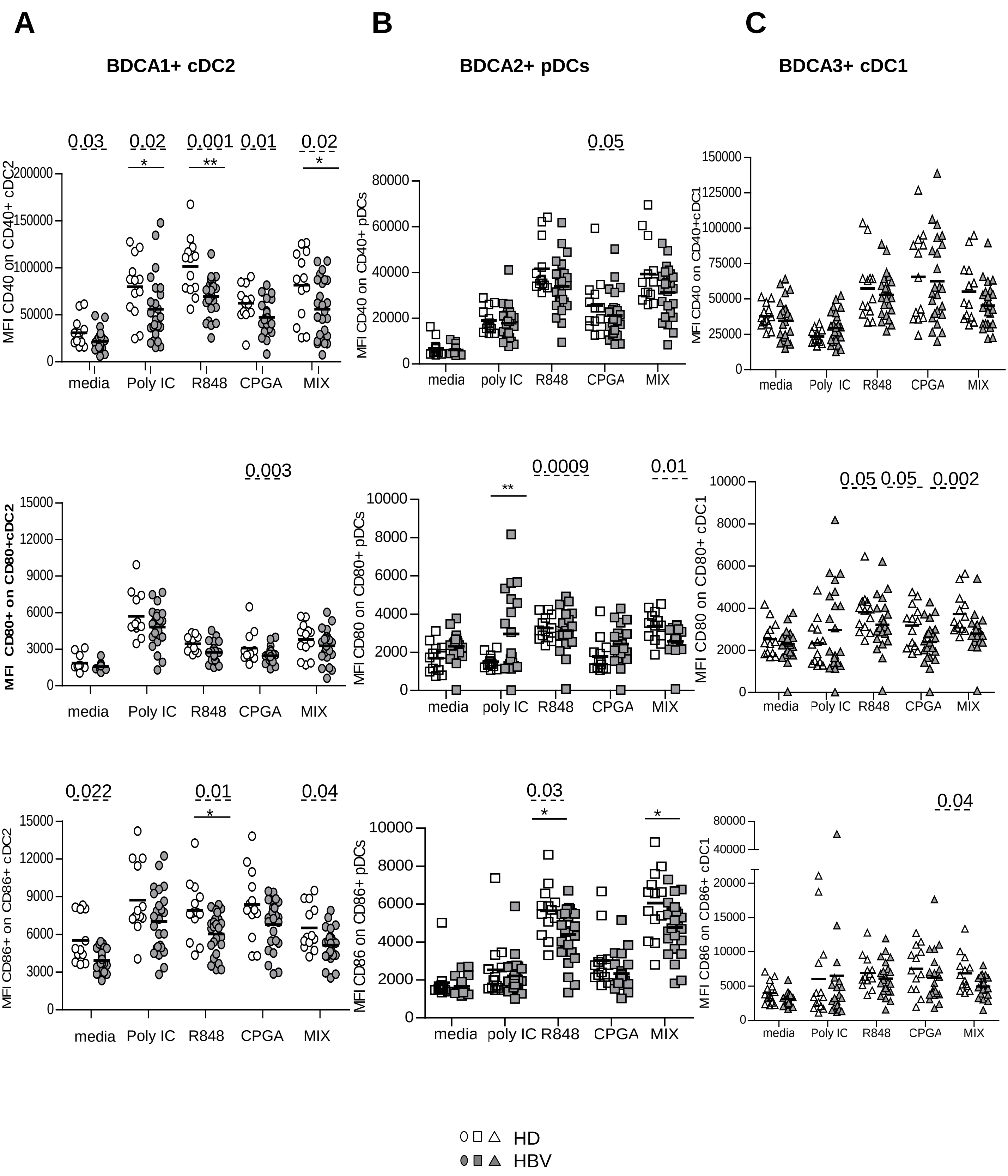

Supporting Figure S6

A **Correlation circulating BDCA1+ cDC2 & BDCA2+ pDCs**

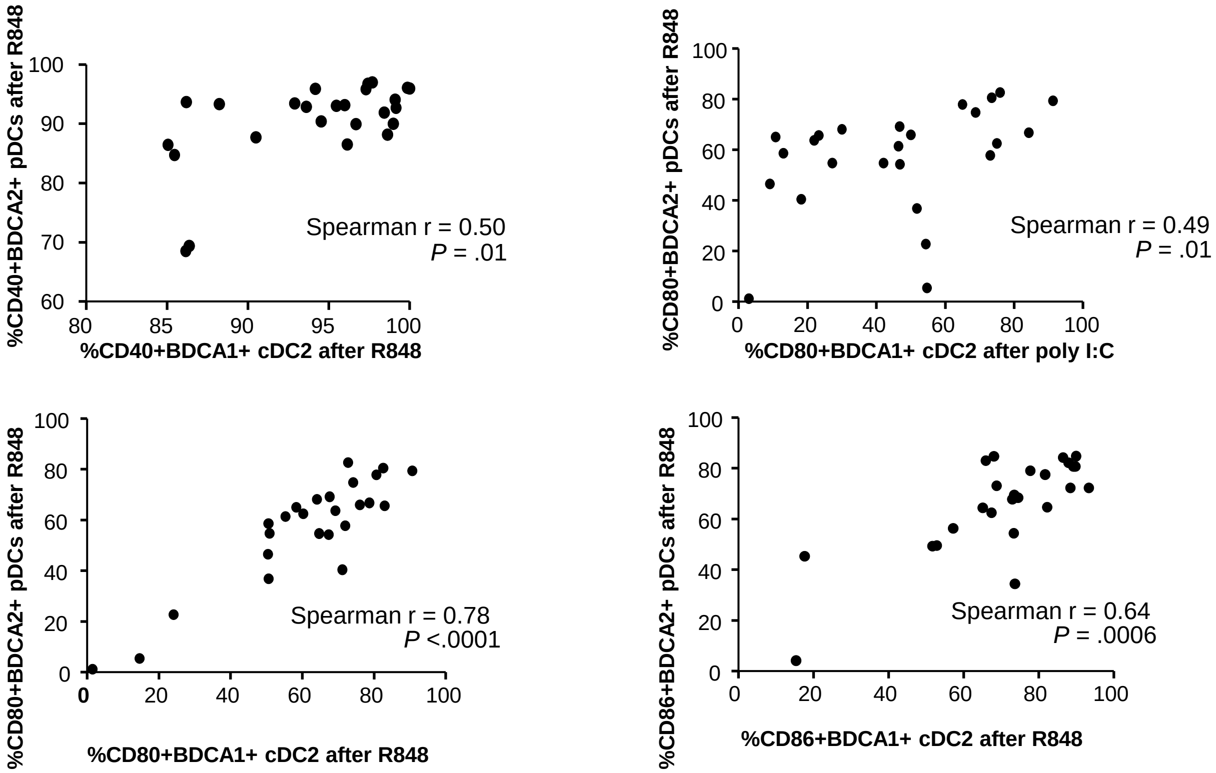

B **Correlation circulating BDCA1+ cDC2 & BDCA2+ pDCs**

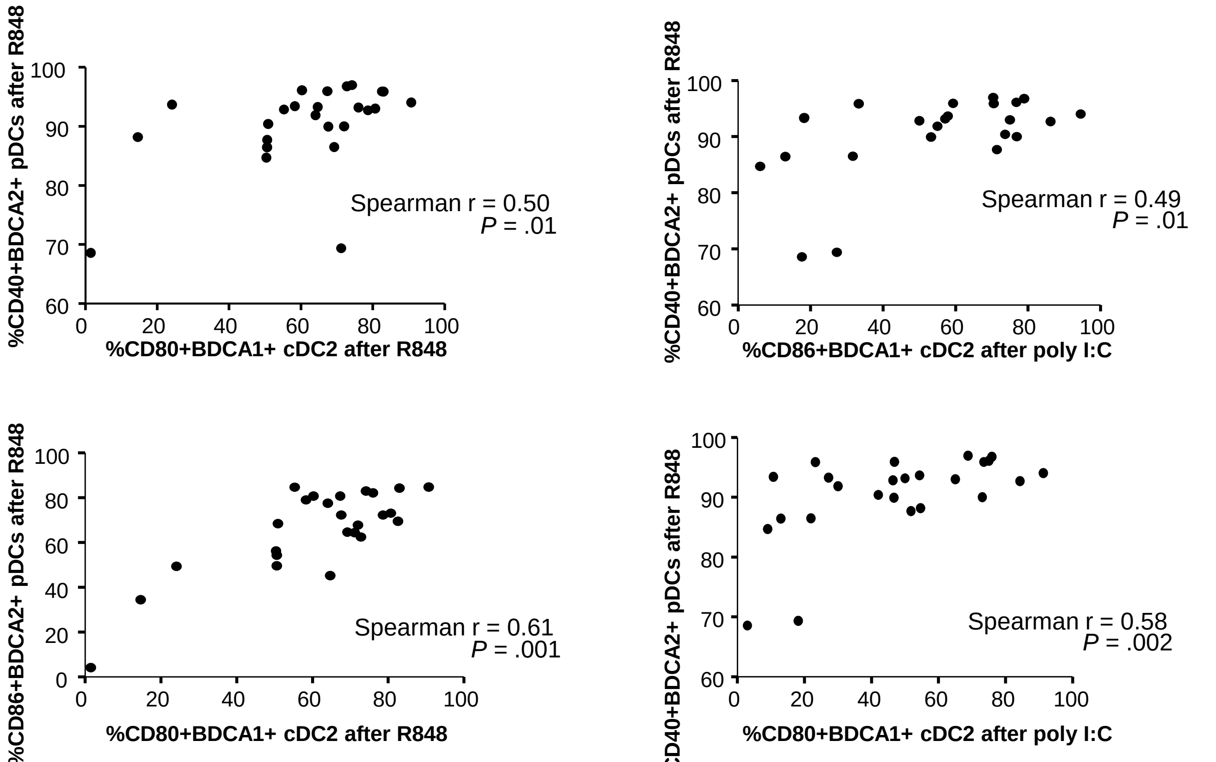

Supporting Figure S7

A

Correlation intrahepatic BDCA1+ cDC2 & BDCA2+ pDCs

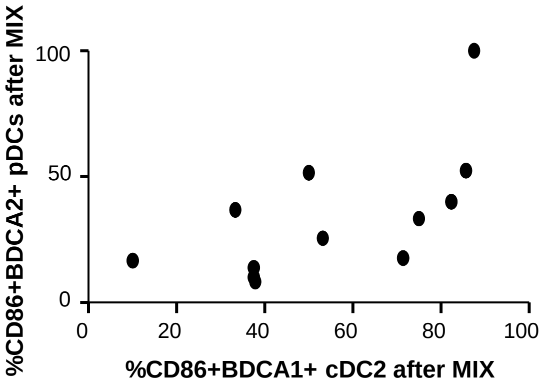

Spearman r = 0.67
P = .02

B

Correlation intrahepatic BDCA1+ cDC2 & BDCA3+ cDC1

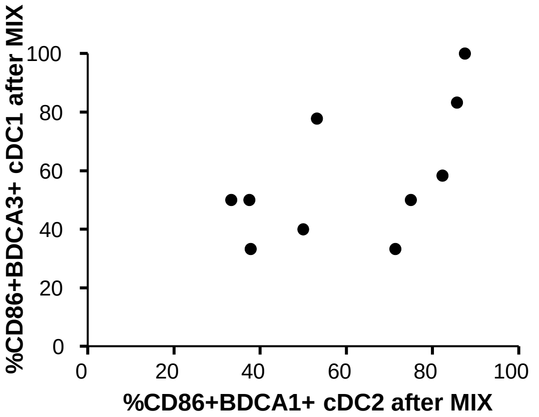

Spearman r = 0.61
P = .058

C

Correlation intrahepatic BDCA3+ cDC1 & BDCA2+ pDCs

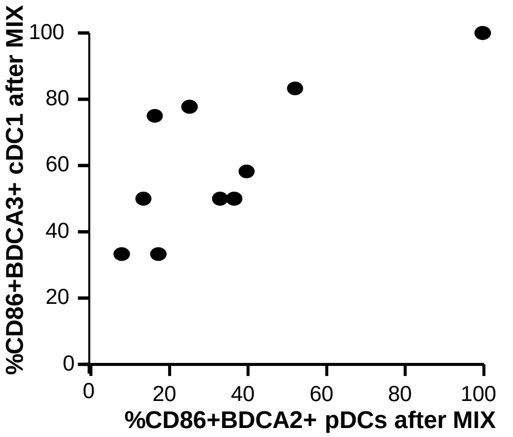

Spearman r = 0.68
P = .03

Supporting Figure S8

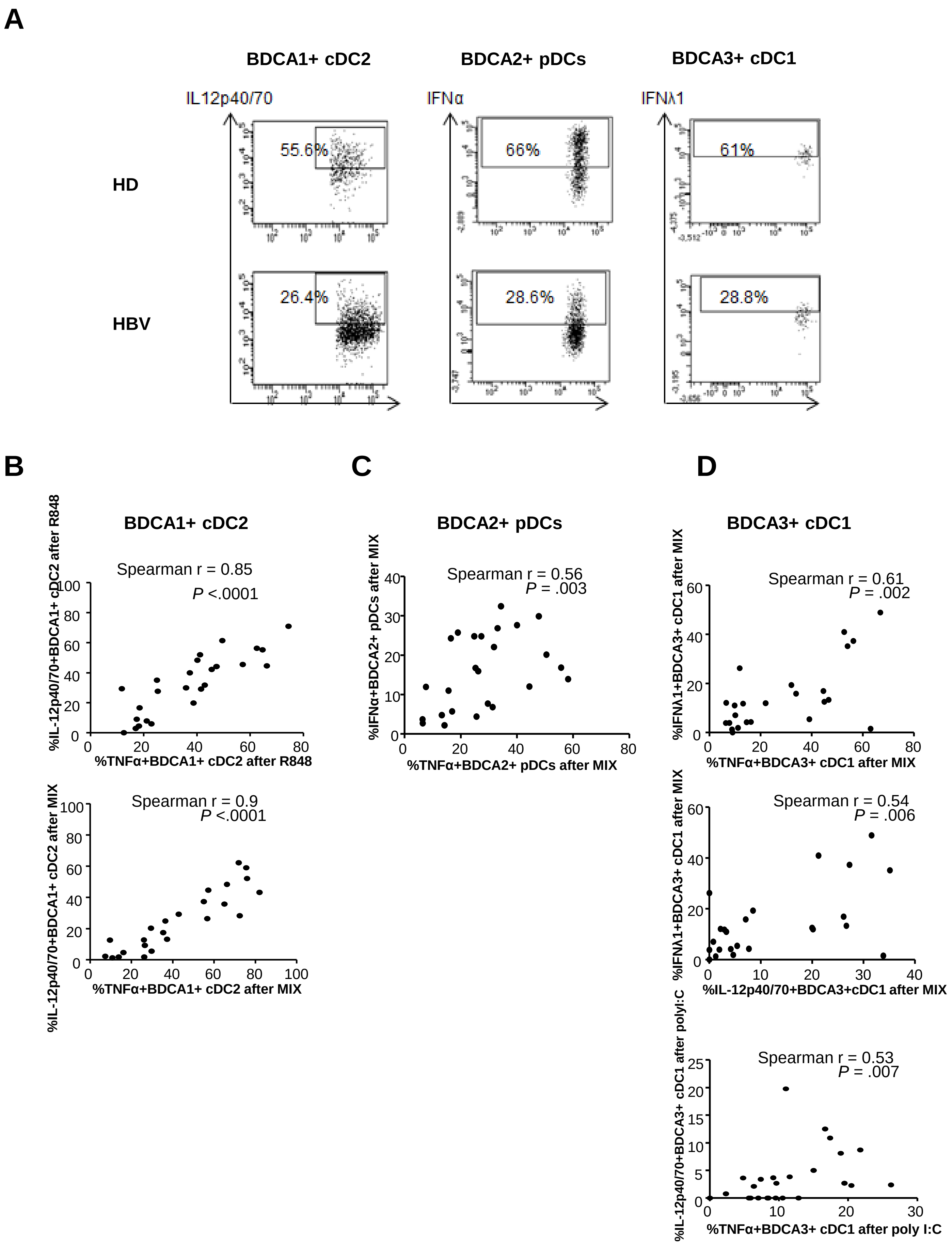

Supporting Figure S9

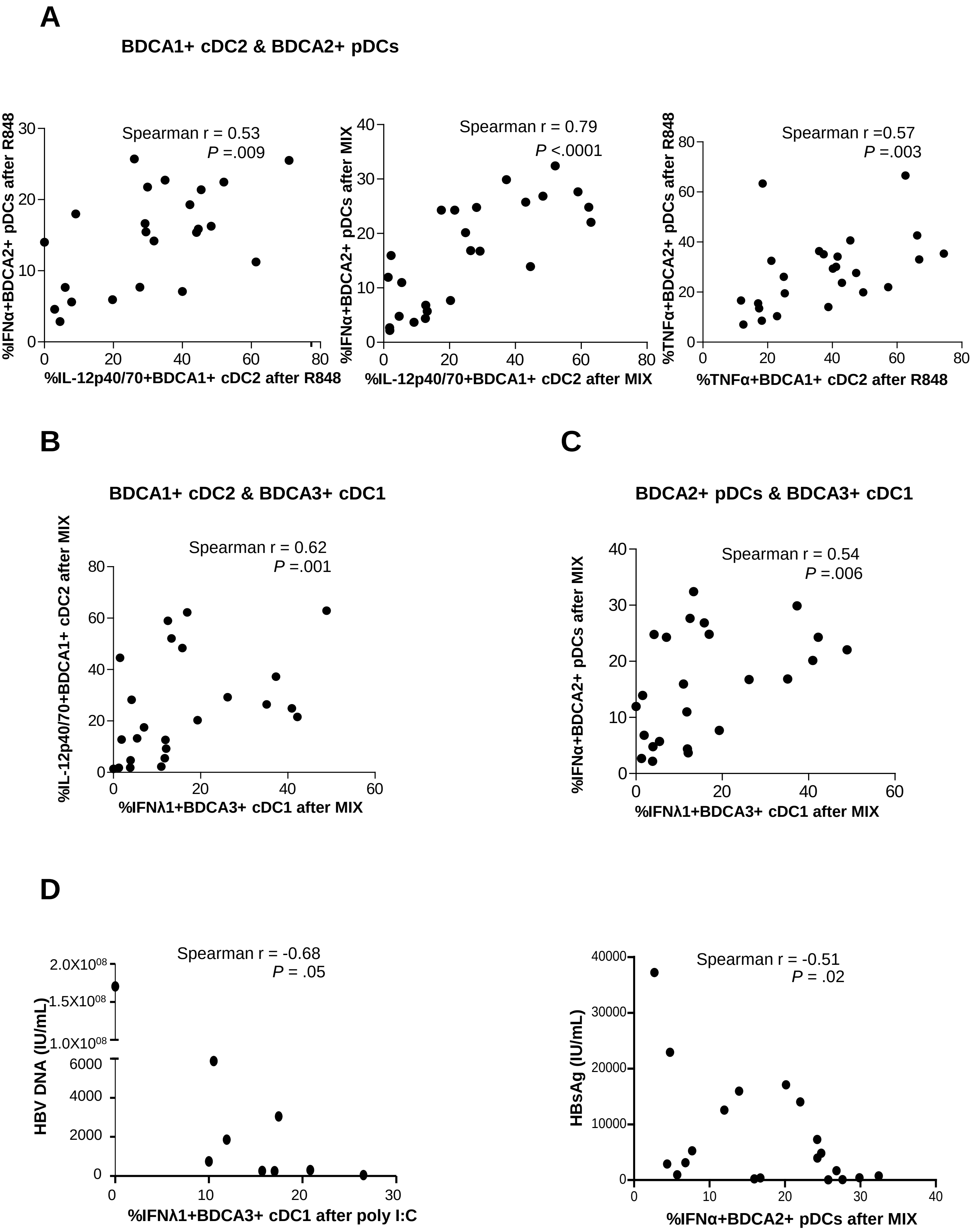

Supporting Figure S10

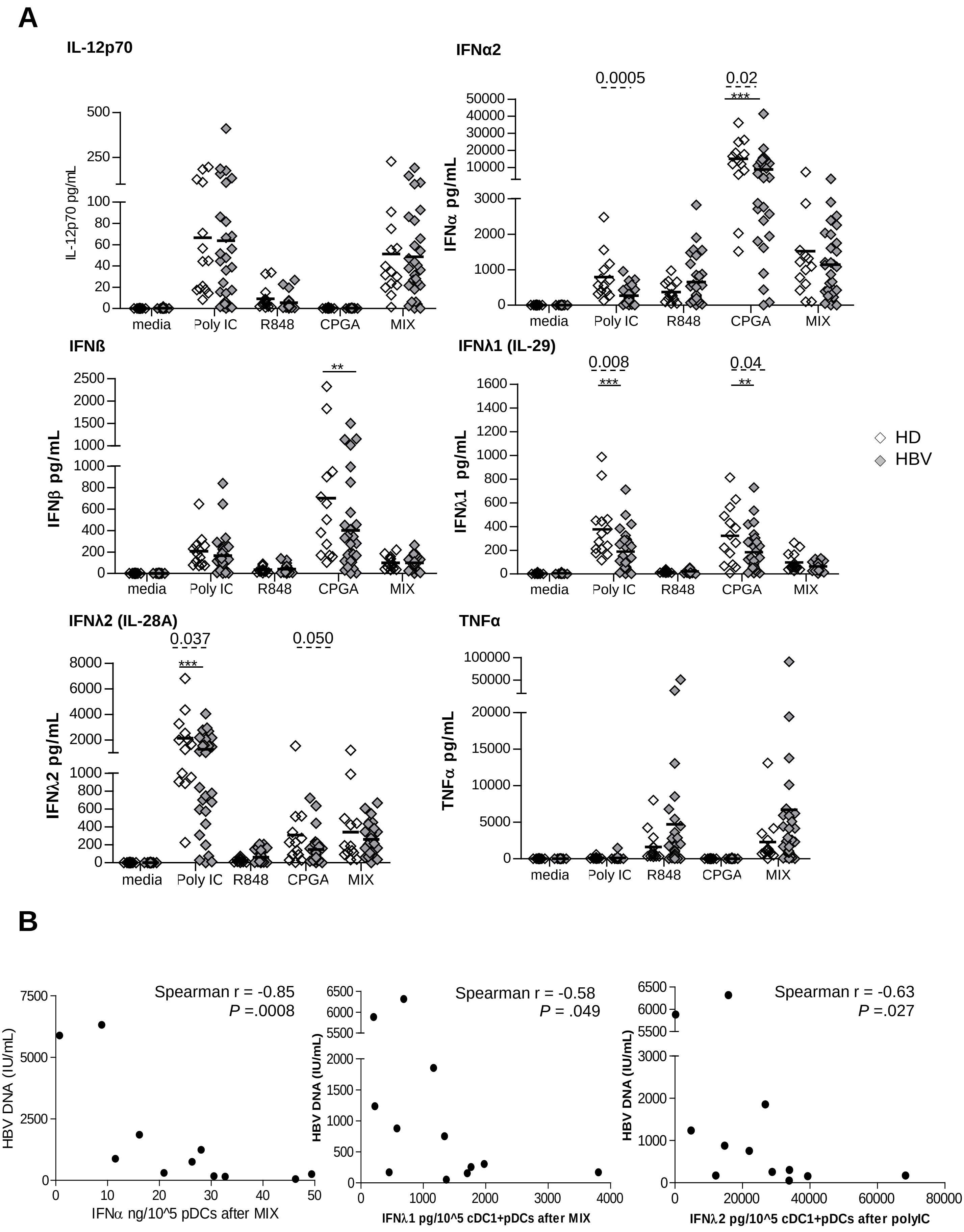

Supporting Figure S11

A

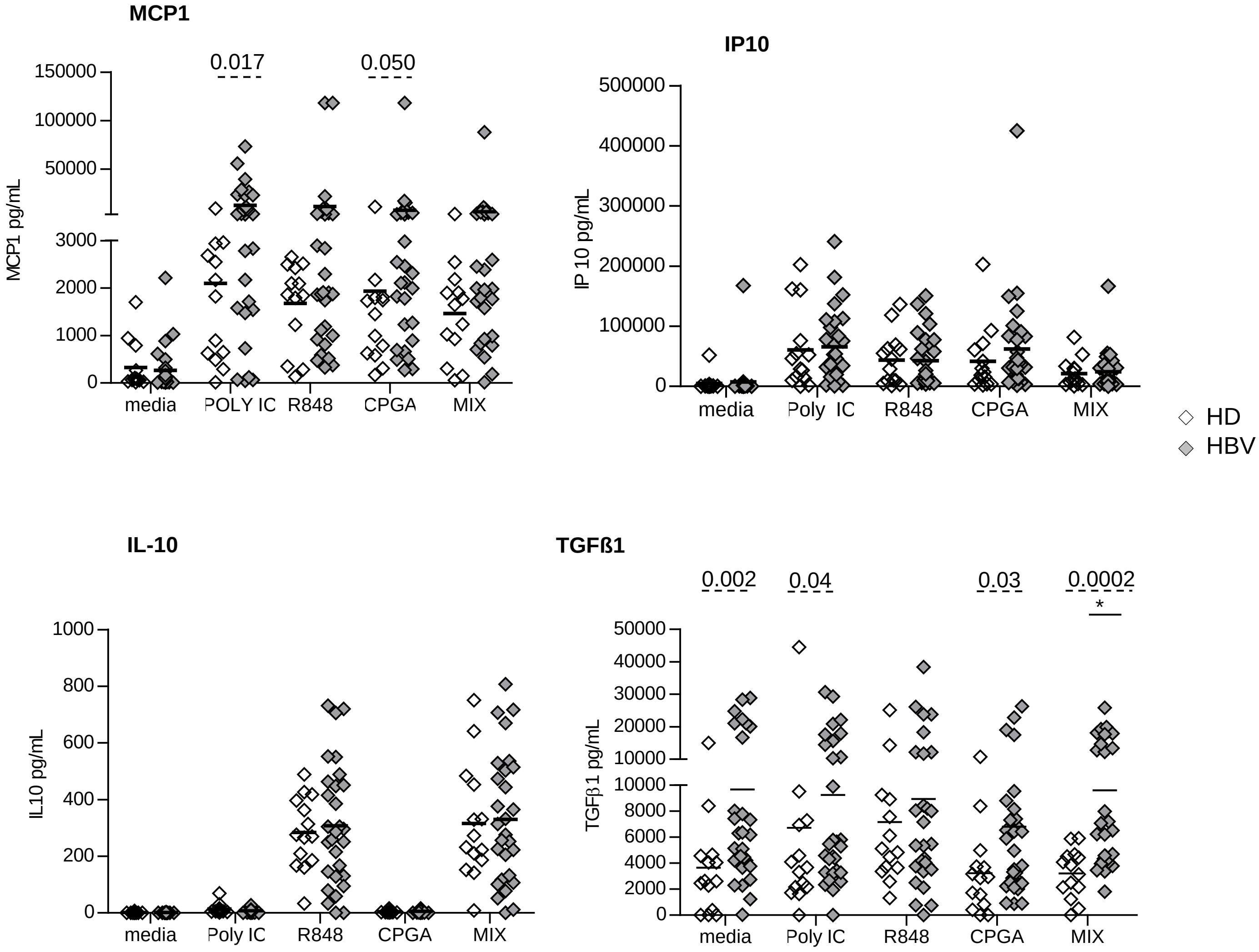

B

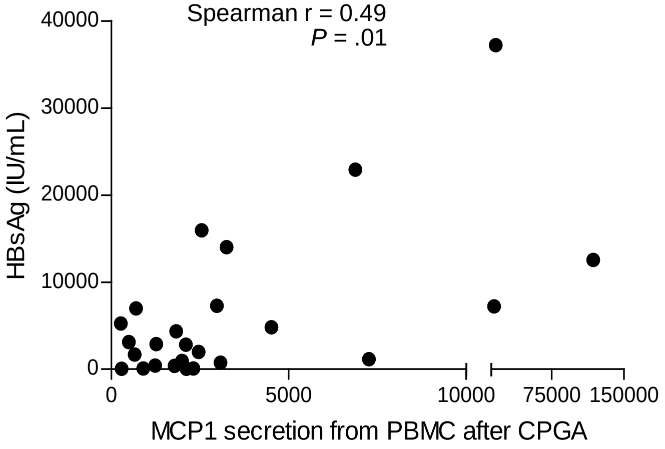

Supporting Figure S12

A

Liver Biopsy

Secreted Cytokine in pg/ml	Biopsy N°1		Biopsy N°2	
	media	MIX	media	MIX
IL12p70	0.66	24.41	0.83	6.64
TNFα	60.7	3363.49	35.84	1629.45
IFNλ2	2.68	72.41	5.35	31.92
IFNβ	2.83	10.16	8.44	15.93
MCP1	198.37	63.23	49.19	5.2
TGFβ1	1716	1042	1601.73	1052.41
IP10	117.72	182.72	31.54	53.49

B

IL-12p70 per cDC1 and cDC2 & IFNλ2 per cDC1 and pDCs

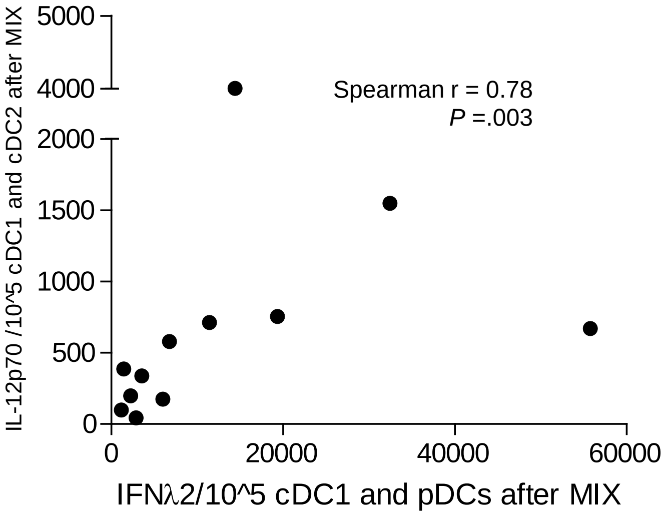

IFNβ per pDCs and IFNλ1 or IFNλ2 per cDC1 and pDCs

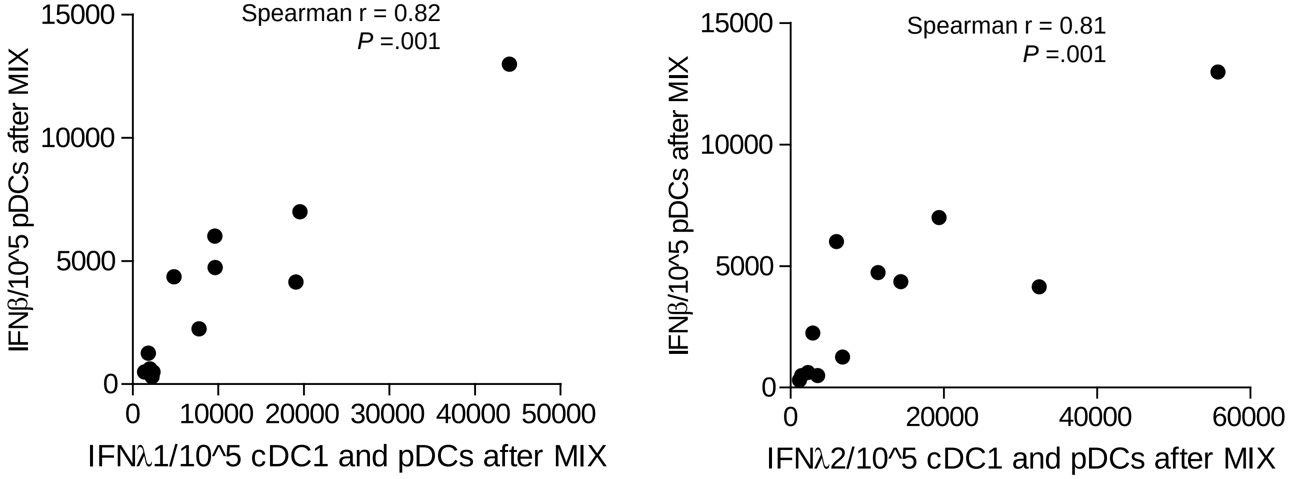

Supplement: Supplementary file 1 [file Data_Sheet_1.PDF]
